# *Lilingostrobus chaloneri* gen. et sp. nov., a Late Devonian woody lycopsid from Hunan, China

**DOI:** 10.1371/journal.pone.0198287

**Published:** 2018-07-11

**Authors:** Philippe Gerrienne, Borja Cascales-Minana, Cyrille Prestianni, Philippe Steemans, Li Cheng-Sen

**Affiliations:** 1 Palaeobiogeology-Palaeobotany-Palaeopalynology, UR Geology, University of Liège, Liège, Belgium; 2 CNRS, University of Lille, UMR 8198 - Evo-Eco-Paleo, Lille, France; 3 OD Earth and Life, Royal Belgian Institute of Natural Sciences, Brussels, Belgium; 4 State Key Laboratory of Systematic and Evolutionary Botany, Institute of Botany, Chinese Academy of Sciences, Beijing, China; Fred Hutchinson Cancer Research Center, UNITED STATES

## Abstract

Lycopsids are a minor component of current terrestrial herbaceous floras. However, lycopsid fossil diversity shows a great diversity and disparity including heterosporous woody plants, e.g. the giant isoetaleans that populated the extensive Pennsylvanian wetlands. The earliest known isoetaleans come from late Devonian localities from China. Here, we describe *Lilingostrobus chaloneri* gen. et sp. nov., a new isoetalean lycopsid from the Upper Devonian (Famennian) Xikuangshan Formation of China (Hunan Province, South China), which adds to the already impressive diversity of the Devonian lycopsids from China. *Lilingostrobus* shows an unusual combination of characters. This new plant is pseudoherbaceous, with a possible tufted habit, and consists of narrow axes with rare isotomies. The stem includes small quantities of secondary xylem. Each fertile axis bears one terminal strobilus comprising sporophylls ending in a very long upturned lamina. Microspores and putative megaspores have been found, but whether the plant has mono- or bisporangiate strobili is unknown. Importantly, our cladistic analysis identifies *Lilingostrobus* as a direct precursor of Isoetales, which provides new insights into the early evolution of lycopsids.

## Introduction

Lycopsids are an early divergent group of vascular plants comprised of two extinct plesions, the Drepanophycales and the Protolepidodendrales, and three extant orders, the isosporous Lycopodiales and the heterosporous Selaginellales and Isoetales, the latter being characterized by secondary growth [1]. With about 1290 extant species [2], lycopsids are a minor component of modern floras. Their evolutionary history is however extremely long: the earliest evidence of Lycopsida are late Ludlow (Late Silurian) specimens of *Baragwanathia* from Australia [3–6]. The Devonian radiation of the lycopsids was spectacular, especially on the South China Block [7–9]. Their diversity markedly decreased during Late Carboniferous in Europe and North America, but they persisted until Permian times in China [10]. Extant lycopsids are small-sized plants.

In extant vegetation, the majority of the arborescent plants belong to the lignophytes that are characterized by a woody stem resulting from the activity of a bifacial vascular cambium, a meristem that produces secondary phloem (inner bark) outwards and secondary xylem (wood) inwards [11]. Secondary growth in lycopsids evolved independently and involves a unifacial cambium that only produces small quantities of secondary xylem; the arborescent habit of the Palaeozoic lycopsids was achieved based on the development of an extensive periderm, which accounted for their impressive trunks.

Lycopsids with secondary growth (wood and periderm) are all heterosporous; they are called Isoetales *sensu* DiMichele and Bateman [12] (= Rhizomorpha *sensu* Bateman [13]). Isoetales are characterized by a bipolar growth from a centralized shoot-like rootstock called the rhizomorph, by stigmarian rootlet formation and by secondary tissue production [12,14]. They are also named “rhizomorphic lycopsids”; *Isoetes* is the only extant genus. The plants included in the Isoetales bear either bisporangiate strobili (producing micro- and megaspores in different sporangia but in the same strobilus) or monosporangiate strobili (producing only one type of spores in a given strobilus). The latter belong to the suborder Dichostrobiles [12]. Most majestic Carboniferous wetland trees were Dichostrobiles; they got extinct at the end of the Palaeozoic.

The earliest heterosporous genera are the Middle Devonian *Mixostrobus* [15], *Yuguangia* [16] and *Longostachys* [17]. *Mixostrobus* [15] and *Yuguangia* [16] bear bisporangiate strobili and do not produce secondary xylem. This character evolved during Middle Devonian times in *Longostachys* [17], and preceded the acquisition of the “monosporangiate strobili” character that defines the Dichostrobiles. Only four Devonian genera with demonstrated monosporangiate strobili are known: *Changxingia* [18,19], *Lepidostrobus* [20], *Minostrobus* [21–24] and *Sublepidodendron* [25–29]. Interestingly, *Sublepidodendron* is the only unambiguous Devonian Dichostrobile, with demonstrated secondary xylem; the internal anatomy of the stem of *Changxingia* and *Lepidostrobus* is yet to be discovered, while only primary growth has been shown in *Minostrobus* [24]. Here we report on *Lilingostrobus* gen. nov., a new Late Devonian small-sized heterosporous lycopsid from China (Liling County, Hunan Province), with well-preserved secondary xylem. *Lilingostrobus* shed additional light on the early evolution of the isoetalean lineage.

## Geological settings

The distribution of Devonian sediments in Hunan Province (China, Fig 1A) includes two areas: The Central-Southern Region (I) and the North-Western Region (II) (see figs 1–17 in Hunan Bureau of Geology and Mineral Resources [30] for details). The top of Upper Devonian in Region I is in turn divided into three units: the Southern Unit, named Jiangyong-Laiyang Unit (I_1_); the Central Unit, Shaoyang-Liling Unit (I_2_) and the Northern Unit, Anhua-Liuyang Unit (I_3_) (Fig 1B).

**Fig 1:**
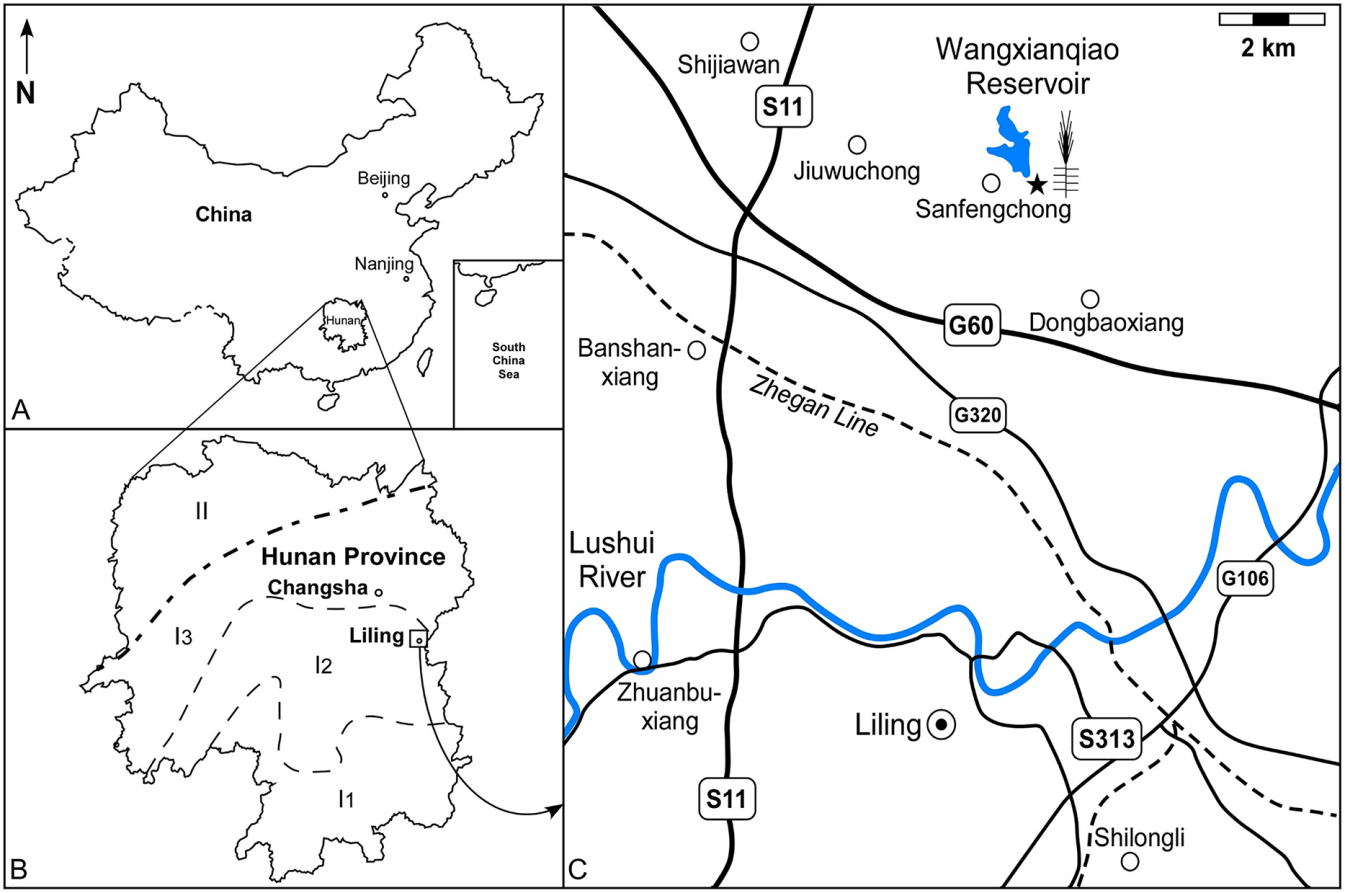
Geographical and geological contexts of the plant-bearing fossil locality. (A) The Hunan Province locates in the South China. (B) The Devonian sediments distribution in Hunnan Province. I: Central-Southern Region; I_1_: Jiangyong-Laiyang Unit, I_2_: Shaoyang-Liling Unit, I_3_: Anhua-Liuyang Unit; II: Northwestern Region. (C) The fossil locality of Wangxianqiao Reservoir is close to the Liling City, Hunana Province. Notes: G, National highway; S, Province highway.

The lithological characteristics of the top of the Upper Devonian succession in Central-Southern Region (I) range from carbonate in the Southern unit (I_1_) to siliciclastic deposits in the Northern Unit (I_3_). More precisely, the Mujingtang Formation in I_1_ and the lower part of the Xikuangshan Formation in I_2_ are composed of carbonate sediments while the upper part of the Xikuangshan Formation in I_2_ and the Yuelushan Formation in I_3_ include siliciclastic deposits. Even though the three formations represent different lithological types, all of them are biostratigraphically characterized by the presence of the age-diagnostic brachiopods *Yunnanella* spp. and *Yunnanellina* spp. which indicate a Famennian (Late Devonian) age.

The fossiliferous layers belong to the Xikuangshan Formation in the northwestern part of Shaoyang-Liling Unit (I_2_) (Fig 1B). The lowermost and uppermost parts of the section were not recovered. The lower part of the section is 77 m thick and consists of limestone, muddy limestone, quartz sandstone, sandstone and shale. The upper part of the section is 106 m thick and includes sandstone, muddy siltstone and sandy shale, with three layers of oolitic hematite purplish red in color close to the top of outcrops. The studied specimens come from muddy, purplish red to grey-yellow siltstone of the upper part of the section. The brachiopods *Yunnanella* sp., *Tenticospirifer* sp., *Cyrtospirifer* sp. and *Camarotoechia* sp. were found from the lower part of the section, while *Lepidodendropsis* sp. and *Sublepidodendron* sp. occur in the upper part [30]. A similar oolitic hematite layer has been also identified in the sediments of Yuelushan Formation in Wufeng Iron Mine at Lianhuaqiao, Changsha County, where the brachiopods *Yunnanella* sp., *Tenticospirifer* sp. and *Cyrtospirifer* sp. occur in the shales, siltstones and quartz sandstones, respectively, above the oolitic hematite layer [31], which provide further support for a Famennian (Late Devonian) age of plant remains.

## Material and methods

### Plant fossil material

The studied specimens were collected in 1983 from the Upper Devonian sediments close to the Wangxianqiao Reservoir (Liling County, Hunan Province, China; Fig 1A), when the dam of the reservoir was repaired. The Wangxianqiao Reservoir is located in the administrative area of Dongbaoxiang (Dongbao Town), in the northern suburb of Liling City (Fig 1B). The recovered plant megafossils are preserved as impression and petrifaction in muddy sandstone. More than 50 fertile specimens were collected. They were studied by using conventional palaeobotanical techniques, including dégagement [32,33], light (LM) and scanning electron microscopy (SEM). Several specimens, including strobili and stems with secondary growth, are three-dimensionally preserved in a very soft and extremely fragile ash-like material. These specimens are most informative, but they are extremely fragile. We tried to embed some of them in order to study them via serial sectioning, but the procedure gave no satisfying results. X-ray computed tomography has been attempted on two specimens at the RBINS (Royal Belgian Institute for Natural Sciences), but gave no satisfying results. SEM was to only efficient way to get detailed information from specimens with this peculiar preservation. It was performed in the CNRS-UMR botAnique et bioinfor Matique de l’Architecture des Plantes (AMAP) of Montpellier (France), and in the laboratory of University of Liege (Belgium) using standard protocols. The fossil material is housed at the repository of the corresponding author´s institution. The collection includes the specimens n° 0901—0902—0903P—0903CP—0904—0906—0907a—0907b—0910—0914—0915—0916—0917—0918—0919. This collection is accessible to external researchers.

### Time-scaled phylogeny

The phylogenetic affinities of *Lilingostrobus* were assessed via a cladistic analysis based on Xue [8]. A data matrix (S1 Table) including 15 of Xue’s [8] core taxa together with *Wuxia* and *Lilingostrobus* (S2 Table) and 33 morphological and anatomical characters (S3 Table) was used. Taxa with too many missing characters such as *Minostrobus* [23] or *Monilistrobus* [34] were not included in the matrix. The inclusion of other taxa such as *Longostachys* [17], *Changxingia* [18] and/or *Paurodendron* [35] resulted in poorly resolved phylogenies and it was decided to reject those taxa. Data analysis was performed using PAUP* 4.0 (Phylogenetic Analysis Using Parsimony, and other Methods) software [36] (S1 Text). The analysis resulted in 9 equally parsimonious trees (Consistency Index (CI) = 0.734; Homoplasy Index (HI) = 0.265; CI excluding uninformative characters = 0.717; HI excluding uninformative characters = 0.282; Retention Index = 0.886; Rescaled consistency index = 0.650). Subsequently, consensus tree topology (S2 Text) was plotted against the stratigraphy in order to construct a time-scaled phylogeny using *strap* (Stratigraphic Tree Analysis for Palaeontology) package [37] of the R statistical software (version 3.2.1, R Developmental Core Team 19 2015) [38]. The calibration of resulting cladogram was performed using the known temporal distribution (S3 Text) of the involved lycopsids (S2 Table, S4 Text). Default options were used but considering a minimum branch length of 1 million years. *strap* analysis was implemented according to the Bell and Lloyd’s tutorial [37] was followed for implementation. See Supporting Information for raw PAUP (S1 Text) and *strap* files (S2 and S3 Text).

### Nomenclature

The electronic version of this article in Portable Document Format (PDF) in a work with an ISSN or ISBN will represent a published work according to the International Code of Nomenclature for algae, fungi, and plants, and hence the new names contained in the electronic publication of a PLOS ONE article are effectively published under that Code from the electronic edition alone, so there is no longer any need to provide printed copies.

The online version of this work is archived and available from the following digital repositories: PubMed Central, LOCKSS.

## Systematic Palaeobotany

**Class**. Lycopsida Kenrick and Crane [1]

**Order and family**. *Incertae sedis*

**Genus**. *Lilingostrobus* gen. nov.

**Diagnosis**. Herbaceous-like plant with isotomously branched axis ending in a compact strobilus. Vegetative leaves persistent, long, acute, with a deep-sunken midvein and spiny margin. Leaves of the widest axes borne in a low helix; leaves on the vegetative portions of the fertile axes borne in pseudowhorls. Exarch, solid primary xylem cylinder, with several peripheral ridges of protoxylem; primary xylem surrounded by a complete layer of secondary xylem including rays. Strobilus composed of a central axis bearing densely placed sporophylls. Presence of micro- and of putative megaspores, but mono- or bisporangiate nature of strobili unknown. Sporophylls disposed in a low helix or in pseudowhorls. Sporophylls consisting of a sub-horizontal proximal portion (pedicel) and a long distal (sub)vertical lamina. Pedicel devoid of alation and of keel. Pedicel bearing one sporangium on its adaxial surface. Sporophyll lamina with trichome-like appendages on its margin. No ligule has been observed.

**Etymology**. Genus name derives from Liling City, near which the specimens were found.

**Type species**. *Lilingostrobus chaloneri* sp. nov.

**Fig 2:**
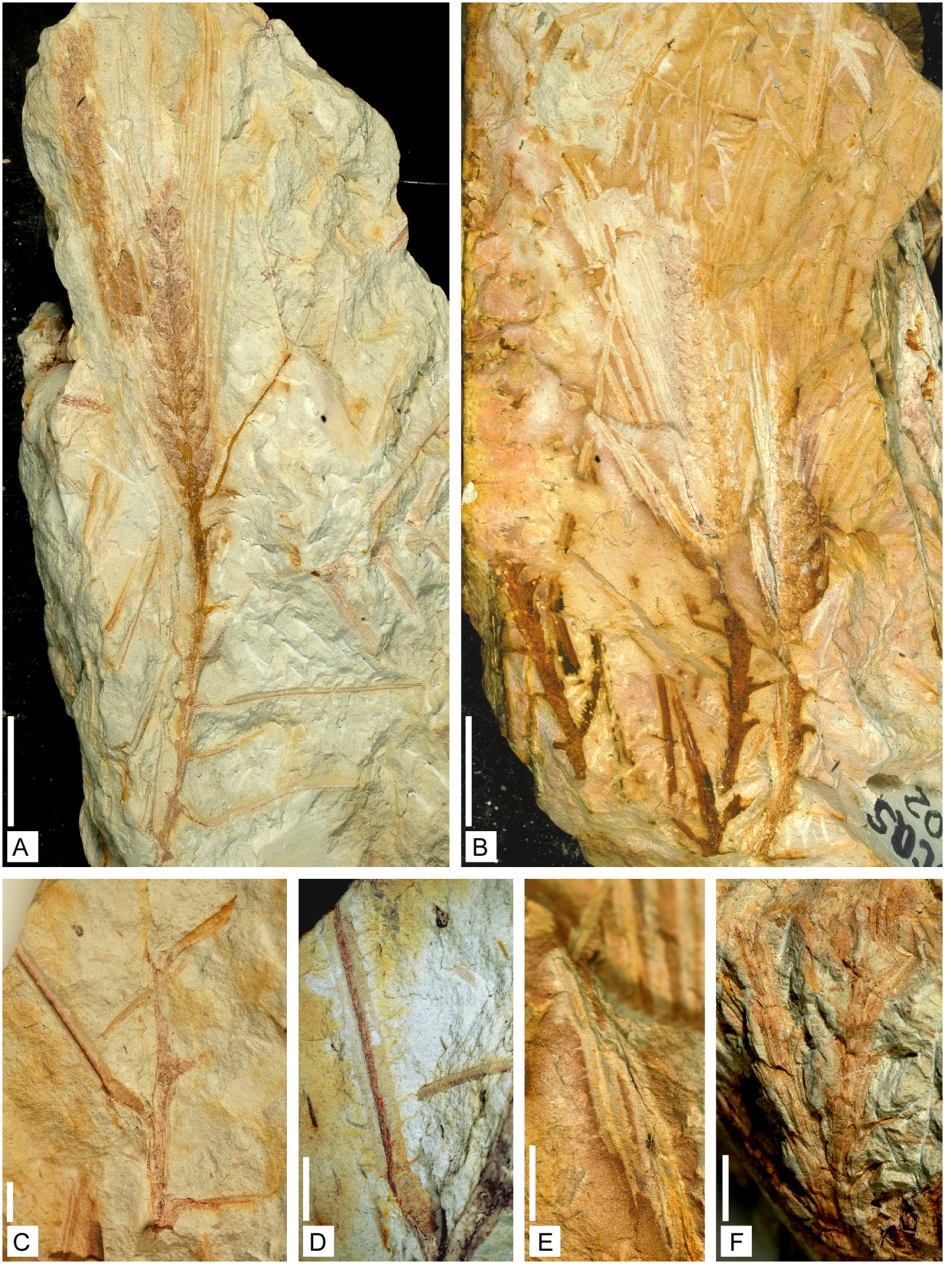
Axes of *Lilingostrobus chaloneri* gen. et sp. nov. (I). (A) Gross lateral view of the holotype. An unbranched stem bearing a distal strobilus. Sterile leaves borne on the stem in a low helix or pseudowhorl. Strobilus with densely arranged long sporophylls. Specimen n° 0901. Scale bar = 1 cm. (B) Lateral view of several stems, more or less parallel to each other. Two strobili are visible. Specimen n° 0902. Scale bar = 1 cm. (C) Lateral view of a stem with well-preserved leaves. Leaf base slightly decurrent. Leaf midvein preserved as a deeply sunken groove. Specimen n° 0907b. Scale bar = 2mm. (D) Detail of C. Vegetative leaf showing trichomes. Specimen n° 0907b. Scale bar = 2 mm. (E) Detail of Fig B. Sporophyll with trichomes. Specimen n° 0902. Scale bar = 2 mm. (F) Gross lateral view of a dichotomous axis. Specimen n° 0906. Scale bar = 1 mm.

**Holotype**. Specimen n° 0901, Fig 2A.

**Repository**. Institute of Botany, Chinese Academy of Sciences, Beijing, China.

**Type locality**. Wangxianqiao Reservoir (Liling County, Hunan Province, China; Fig 1).

**Horizon**. Upper Devonian (Famennian) Xikuangshan Formation.

**Etymology**. The species is dedicated to Professor William Chaloner, in recognition of his outstanding contribution to Palaeobotany.

**Diagnosis**. Width of axis ranging from 1.5 mm to 5 mm. Leaf pseudowhorls 3–5 mm apart. Vegetative leaves slightly decurrent, inserted at 45–90° on the axis. Leaf at least 30 mm long and up to 1.7 mm wide. Leaf margin bearing trichome-like appendages, up to 1 mm long and 0.1 mm wide in their proximal part, 5–10 mm apart along the leaf margin. Primary xylem strand 1.0 to 1.8 mm across, with 8–12 exarch protoxylem strands. Metaxylem cells rounded in transverse section, 20–60 μm in diameter; presence of Williamson’s striation. Protoxylem cells 7–20 μm in diameter. Secondary xylem tracheids 30–50 μm in diameter. Rays possibly more than 100 cells high, including approximately rectangular thin-walled, presumably parenchymatous, cells, 20–50 μm high and 50–100 μm long. Tracheid/ray (cross-field) pitting consisting of ca. 20 rounded to oval pits, 5–10 μm high and wide. Strobilus up to 56 mm long, and up to 7 mm wide. Sporophylls up to 45 mm long and 1.0–1.6 mm wide, inserted on the strobilus axis at 45–90°. Sporophyll pedicel approximately 2.0 to 3.5 mm long and 0.2–0.4 mm wide; distal lamina up to 50 mm long. Trichome-like appendages borne on leaf margin, up to 0.5 mm long. Putative sporangia globose, 1–2 mm high and wide, possibly attached on a short stalk inserted distally on the pedicel of the sporophyll. Microspore around 50 μm in diameter, with subcircular amb. Trilete mark extending to the amb radius. Curvaturae possibly present. A slightly prominent triangular area with concave sides is present at the proximal pole. In the interradial and proximo-equatorial area, one specimen shows small parallel rugulae of 4–5 μm thick and apart, and 20–30 μm length. Distal face smooth. Putative megaspores 300–350 μm in diameter.

## Description

**Fig 3:**
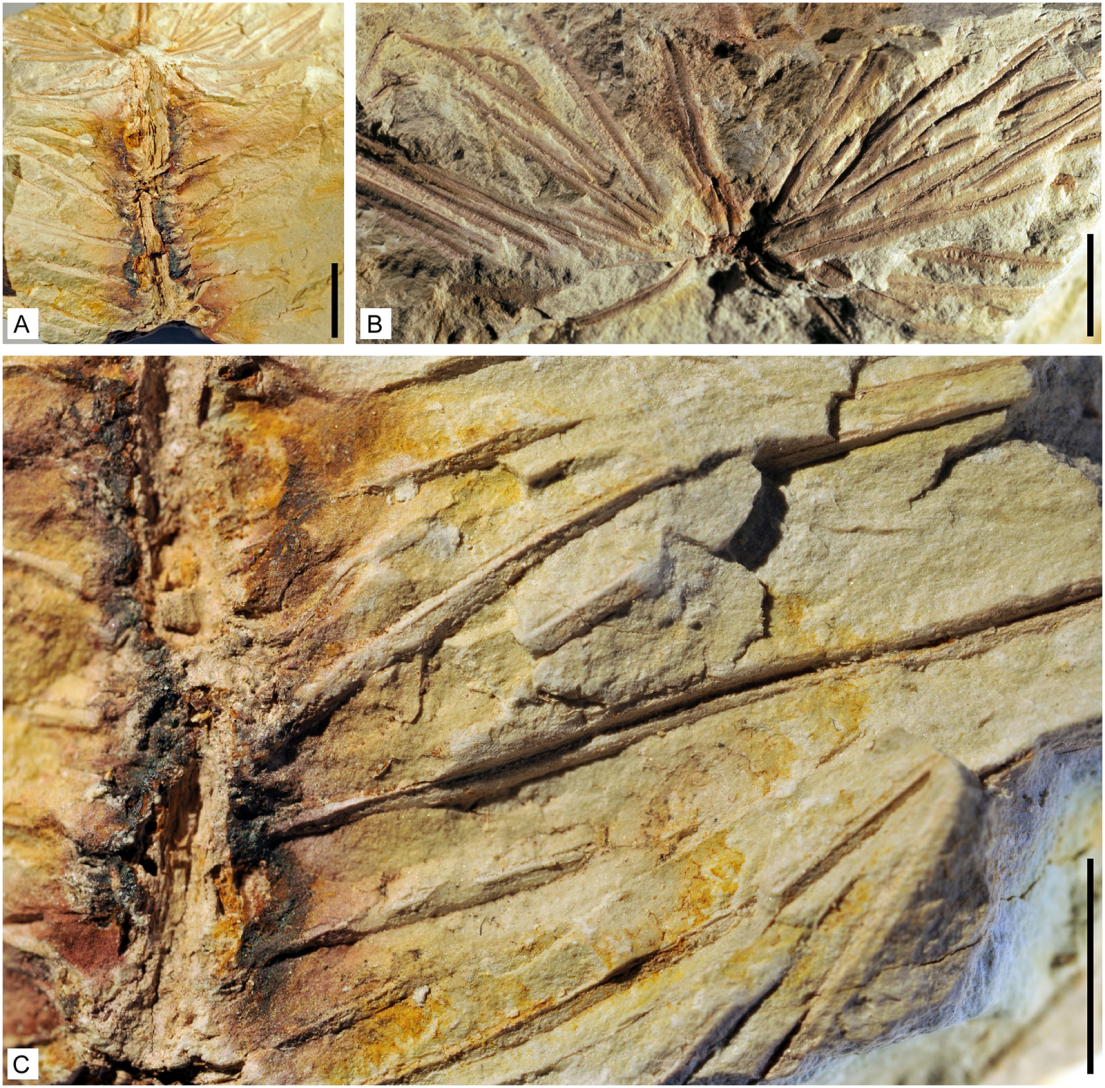
Axes of *Lilingostrobus chaloneri* gen. et sp. nov. (II). (A) Stem with proximal and distal extremities broken. Vegetative leaves borne on the stem in a low helix or pseudowhorl. Specimen n° 0903P. Scale bar = 1 cm. (B) Detail of pseudowhorls showing more than 5 leaves per pseudowhorl. Deeply sunken grooves indicating leaf midveins. Specimen n° 0903CP. Scale bar = 5 mm. (C) Enlargement of Fig 3A showing the leaves and a partially petrified stem. Specimen n° 0903P. Scale bar = 5 mm.

**Fig 4:**
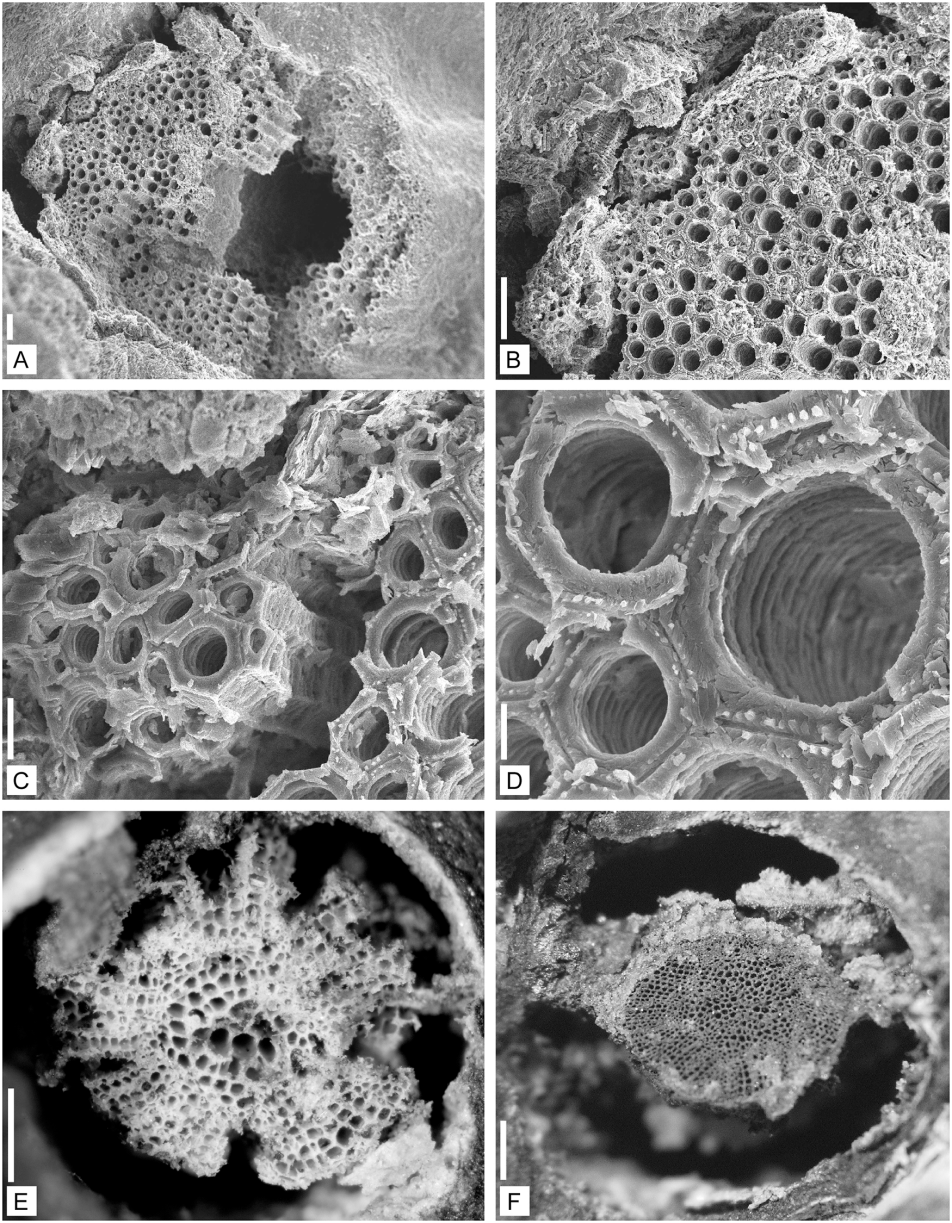
Anatomy of the vascular strand of *Lilingostrobus chaloneri* gen. et sp. nov. (I). Figs A–D. Specimen n° 0903. (A) SEM of a transverse section of primary xylem bundle preserved in three-dimension. Scale bar = 100 μm. (B) Enlargement of Fig A. SEM of the protoxylem strands at the margin of primary xylem cylinder, with possible oval leaf traces (an arrow) separating from the primary xylem cylinder halfway between two protoxylem strands. Scale bar = 100 μm. (C) Enlargement of Fig B. SEM of a leaf trace preserved in three-dimension. Scale bar = 20 μm (D) Enlargement of Fig B. SEM of three tracheids. Scale bar = 10 μm (E) LM of the primary and secondary xylem preserved in three-dimension, seen in transverse section. Primary xylem is located in the central portion of xylem cylinder while the secondary xylem is arranged radially in the peripheral region. Specimen n° 0910. Scale bar = 1 mm. (F) LM of the primary and secondary xylem is preserved in three-dimension in transverse section. Primary xylem is located in the central portion of xylem cylinder while the secondary xylem is arranged radially in the peripheral region. Specimen n° 0915. Scale bar = 1 mm.

**Fig 5:**
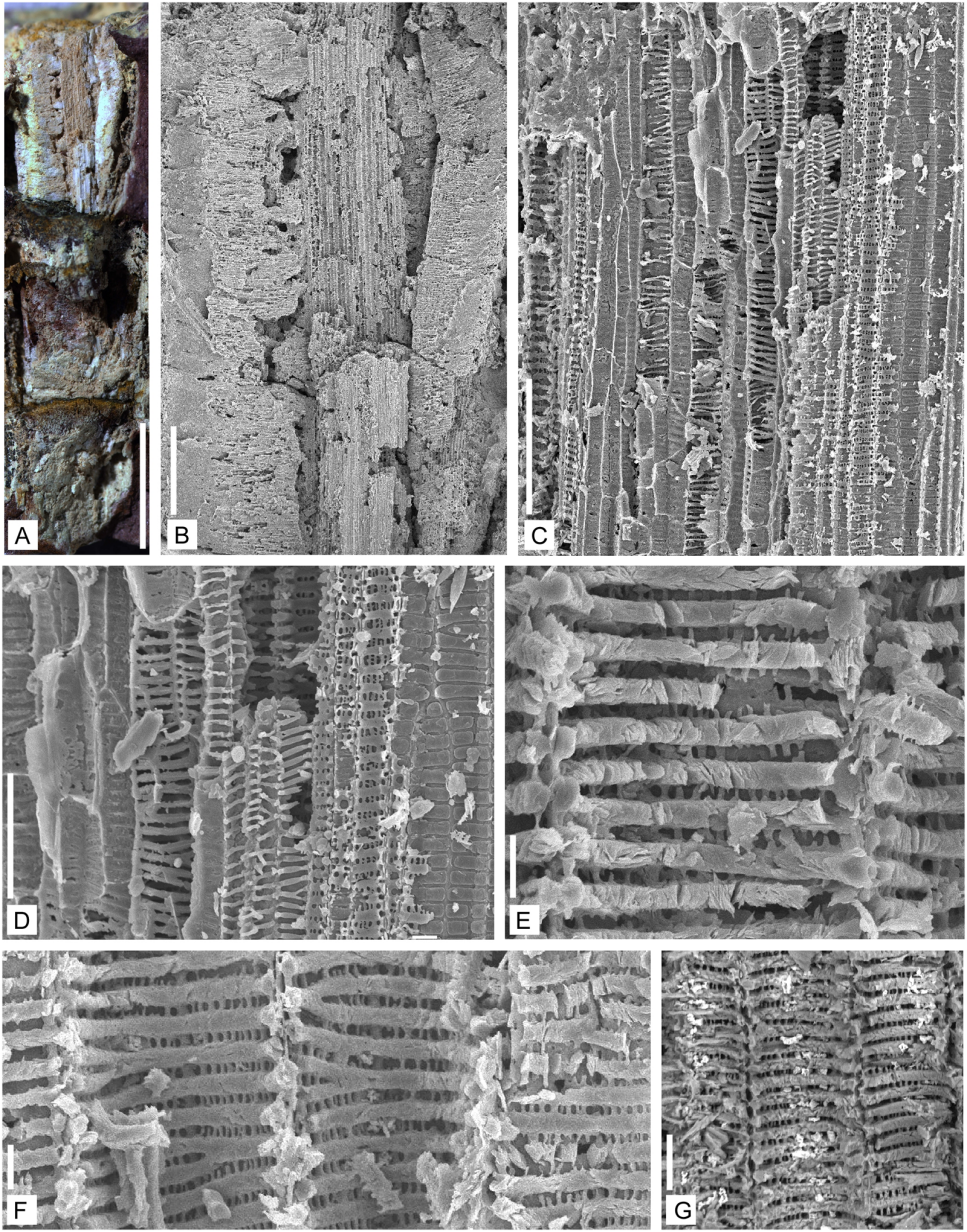
Anatomy of the vascular strand of *Lilingostrobus chaloneri* gen. et sp. nov. (II). Figs A–G. Specimen n° 0914. (A) LM of a portion of a stem showing leaves broken distally and a vascular strand (upper part). Scale bar = 5mm. (B) SEM of a longitudinal view of vascular strand. Primary xylem is in the central portion while secondary xylem is located laterally. Scale bar = 1 mm. (C) Enlargement of Fig B. SEM of the protoxylem tracheids (central part of the picture) and the metaxylem tracheids (left and right), as well as probable vertical parenchyma. Scale bar = 100 μm. (D) Enlargement of Fig B. SEM of the annular/helical thickening of protoxylem tracheids (central part of the picture) and scalariform thickenings of the metaxylem tracheids in the adjacent areas. Scale bar = 50 μm. (E–G) Enlargement of Fig A. SEM of tracheids showing the scalariform pitting, pit aperture and Williamson’s striations (vertical and narrow fibrils connecting two successive scalariform thickening bars) of the metaxylem tracheids. Scale bar = 10 μm in E and F; 20 μm in G. Note: the horizontal thickening bars appear completely filled with amorphous material. Some horizontals bars are branched.

**Fig 6:**
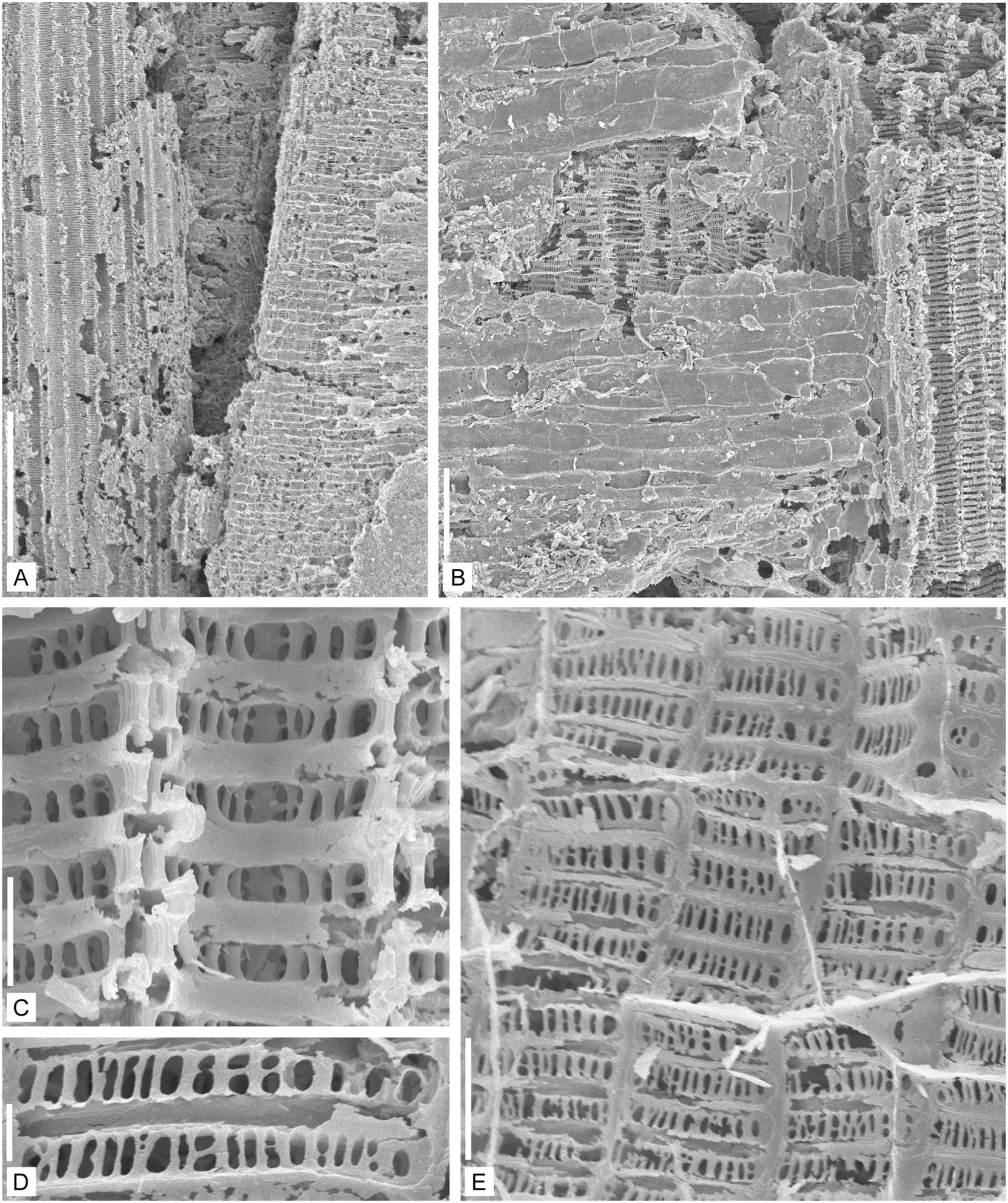
Anatomy of the vascular strand of *Lilingostrobus chaloneri* gen. et sp. nov. (III). (A–E) SEM of specimen n° 0914 in longitudinal views. Enlargment of Fig 5A and 5B. (A) Tracheids of the metaxylem (left) and secondary xylem (right). Scale bar = 500 μm. (B) Metaxylem tracheid (right) and secondary xylem tracheid (left), the latter partly covered by ray(s). The rays consist of horizontally disposed, rectangular, thin-walled, parenchymatous cells. Scale bar = 100 μm. (C) Scalariform bars of tracheids of the secondary xylem and the Williamson’s striations. Note that the horizontal thickening bars are hollow. Scale bar = 10 μm. (D–E) Cross-field (tracheid/ray) pitting including numerous oval to rounded pitlets. In E, the outlines of the ray parenchymatous cells are visible. Scale bar = 10 μm in D and 20 μm in E.

**Fig 7:**
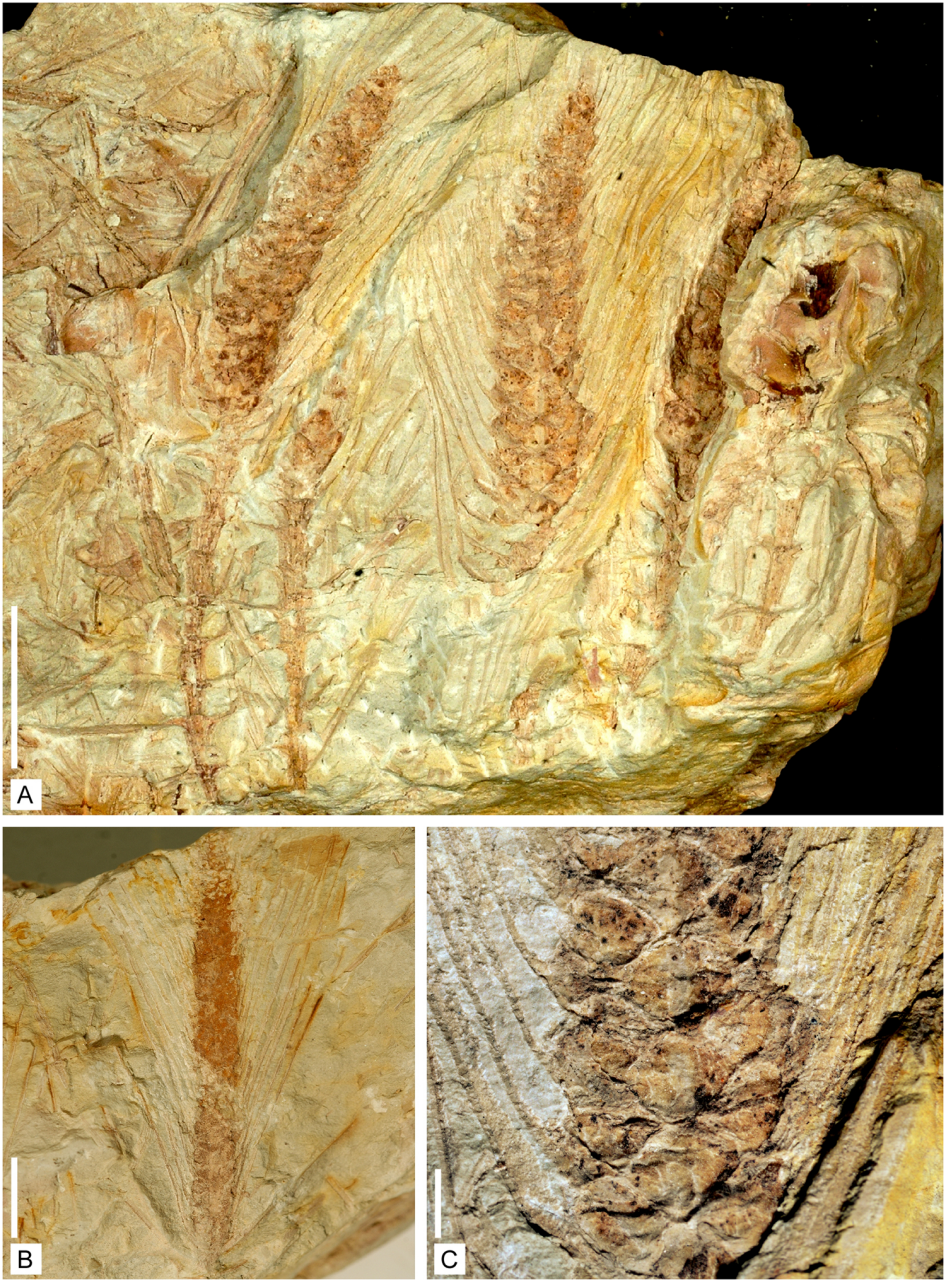
Reproductive structures of *Lilingostrobus chaloneri* gen. et sp. nov. (I). (A) Gross view of several parallel-sided stems suggesting that they are preserved nearly *in situ* with several strobili are visible. Specimen n° 0904. Scale bar = 1 cm. (B) Isolated fertile specimen showing the densely placed, parallel subvertical laminae of sporophylls. Specimen n° 0906. Scale bar = 1 cm. (C) Detail of Fig A. showing the arrangement of sporophylls on the fertile axis and the base of sporophyll lamina. Specimen n° 0904. Scale bar = 2 mm.

**Fig 8:**
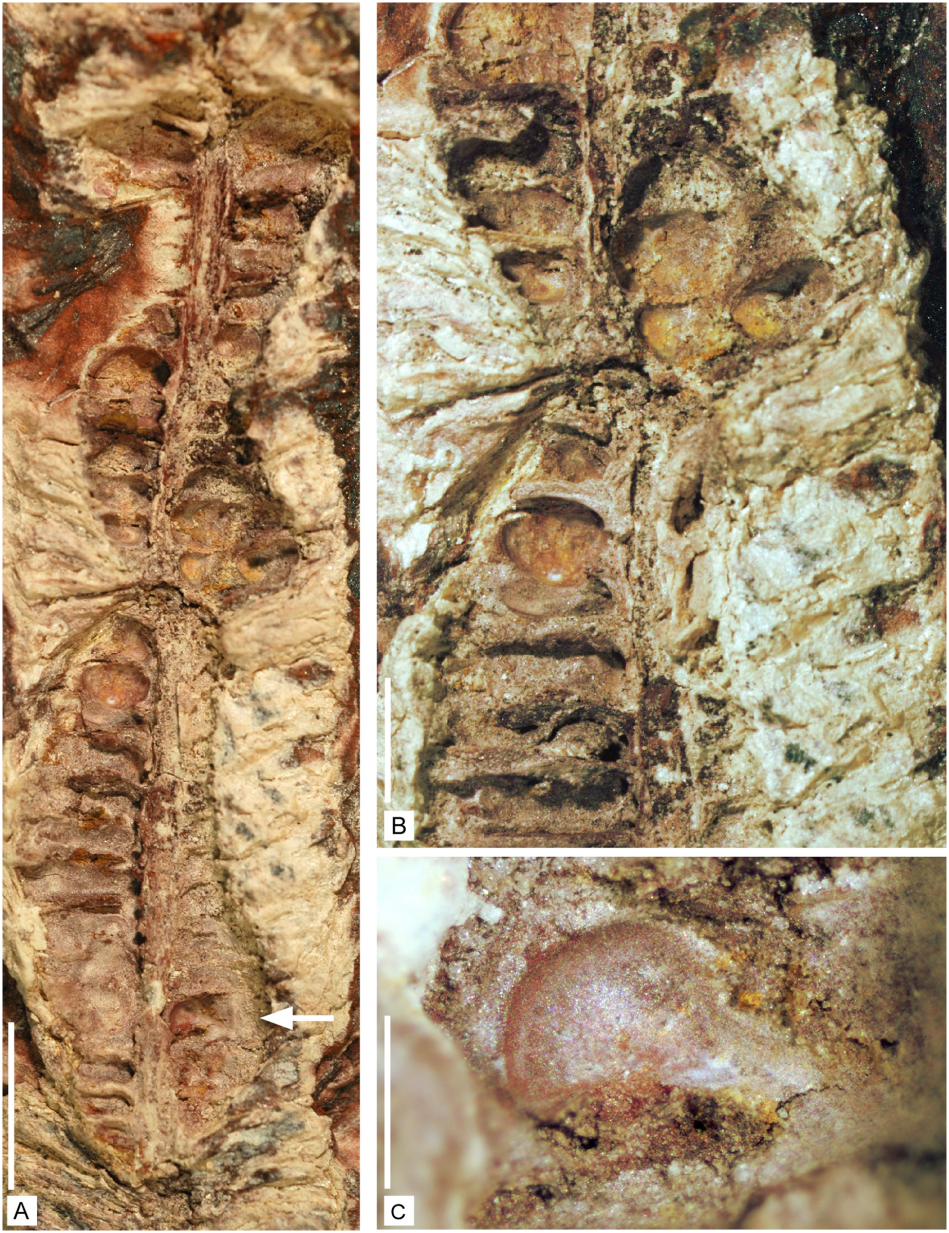
Reproductive structures of *Lilingostrobus chaloneri* gen. et sp. nov. (II). (A) Strobilus preserved as a three-dimensional cast. The pedicels of the sporophylls are inserted on fertile axis at an angle of 90 degrees and sporangia are located in between pedicels. Specimen n° 0919. Scale bar = 5 mm. (B) Enlargement of the middle part of Fig A showing the rounded bodies interpreted as sporangia. Specimen n° 0919. Scale bar = 2 mm. (C) Enlargement of the lower part of Fig A. Arrow indicates a possible rounded sporangium with a short stalk attached at the angle between pedicel and sporophyll lamina. Specimen n° 0919. Scale bar = 1 mm.

**Fig 9:**
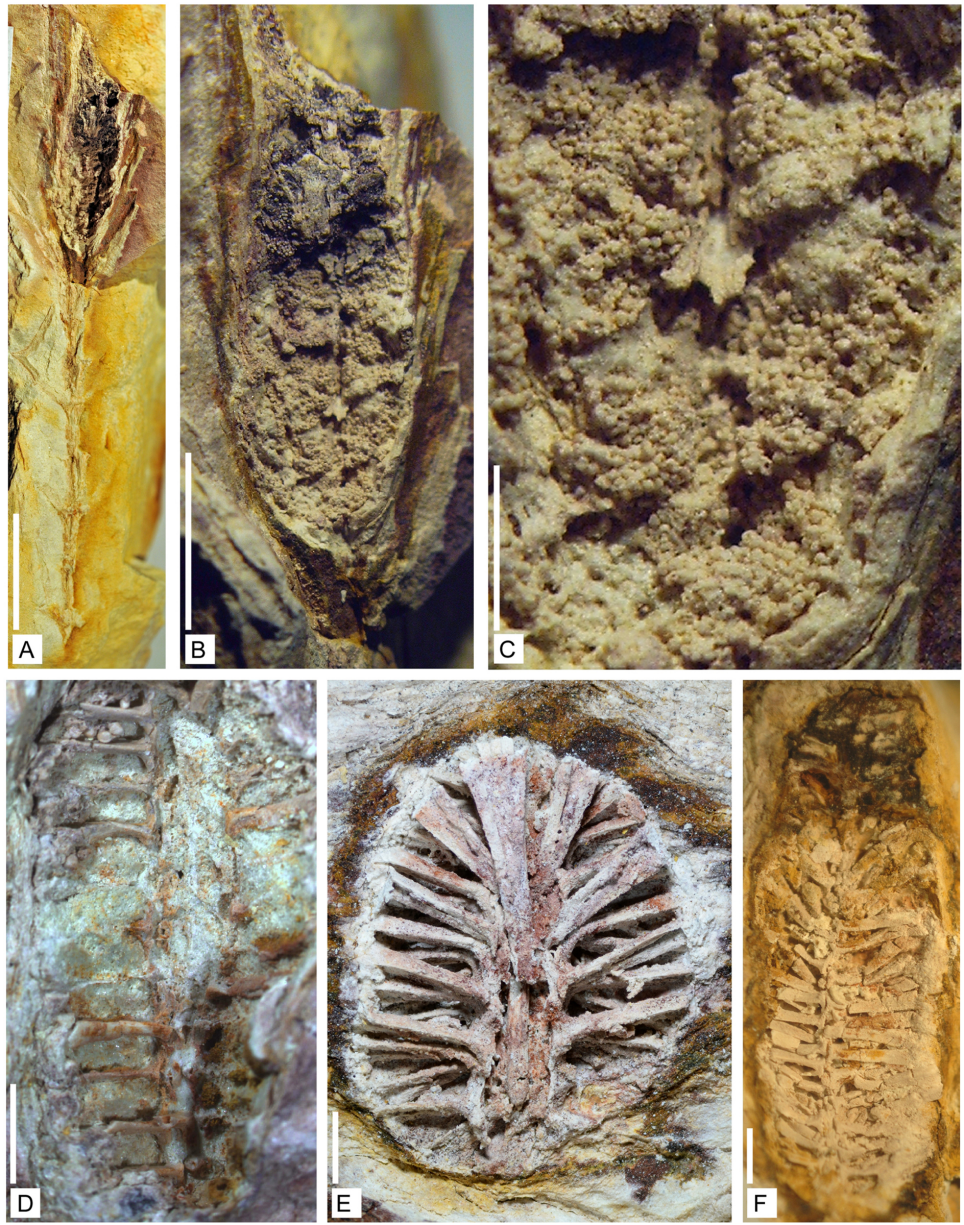
Reproductive structures of *Lilingostrobus chaloneri* gen. et sp. nov. (III). (A) Strobilus with microspores. Specimen n° 0907a. Scale bar = 1 cm. (B) Enlargement of Fig A. showing the strobilus with numerous microspores (white cast). Specimen n° 0907a. Scale bar = 5 mm. (C) Enlargement of Fig B. showing the three-dimensional microspore casts. Specimen n° 0907a. Scale bar = 1 mm. (D) Strobilus preserved as a three-dimensional cast showing putative megaspores at the left top. Pedicels of sporophylls inserted on the fertile axis at an angle of 90 degrees. Specimen n° 0916. Scale bar = 2 mm. (E) Strobilus preserved as a three-dimensional cast. The pedicels of sporophylls are inserted on the fertile axis at an acute angle. Specimen n° 0918. Scale bar = 2 mm. (F) Strobilus preserved as a three-dimensional cast. Specimen n° 0917. Scale bar = 2 mm.

**Fig 10:**
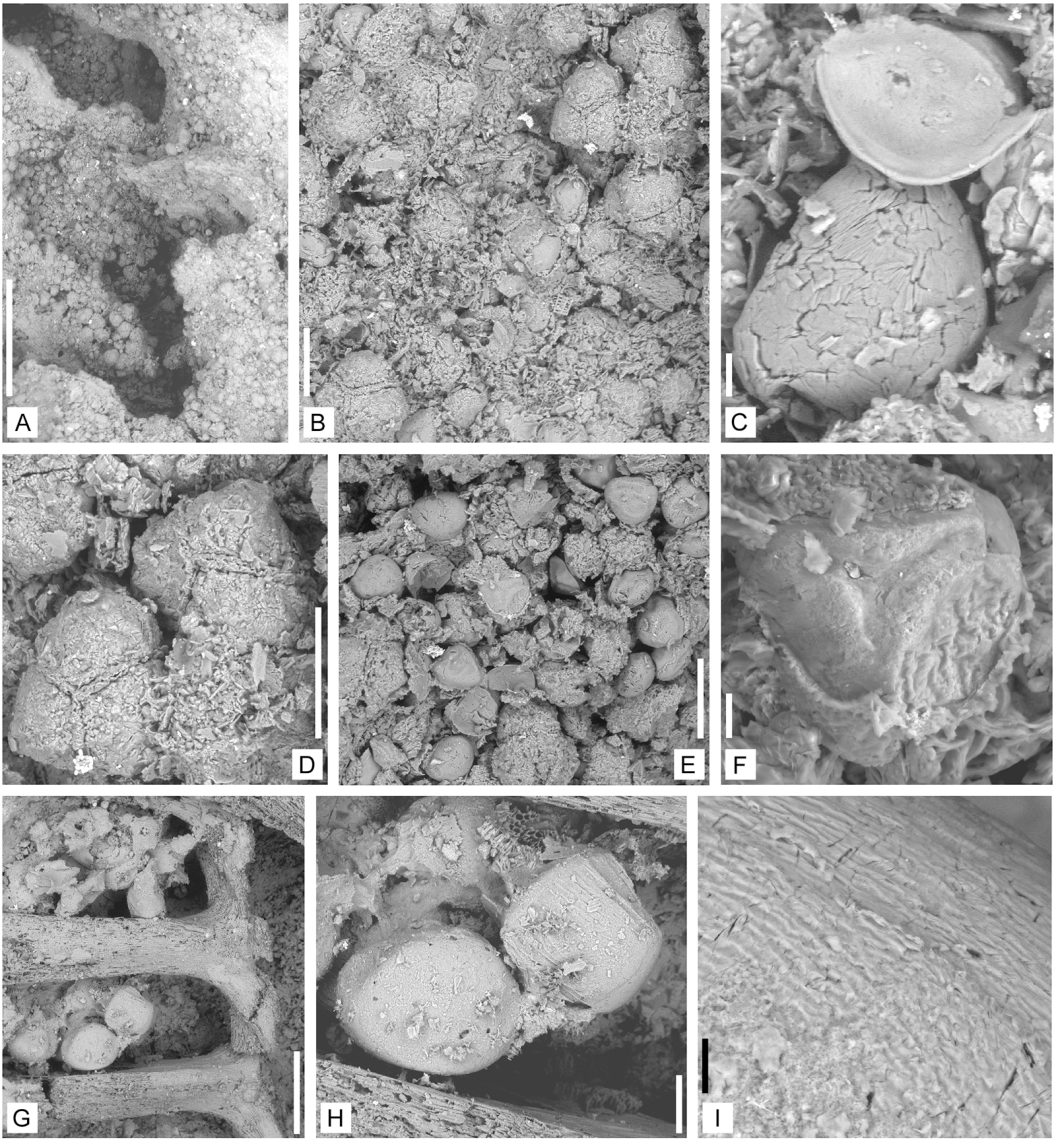
Micro- and putative megaspore morphologies of *Lilingostrobus chaloneri* gen. et sp. nov. Figs. A–F. Specimen n° 0907a viewed under SEM. (A) Gross view of microspores preserved in situ. Scale bar = 1 mm. (B) Tetrads of microspores preserved as cast. Scale bar = 100 μm. (C) Microspores preserved as casts (?). Scale bar = 10 μm. (D) Enlargement of Fig B showing tetrads of microspores. Scale bar = 100 μm. (E) Mass of microspores preserved in situ. Scale bar = 100 μm. (F) Enlargement of Fig E showing a spore trilete mark. Scale bar = 10 μm. Figs. G–I. Specimen n° 0916. (G) Portion of the strobilus from Fig 9D showing putative megaspores, which are preserved in between the pedicels in situ in three-dimension. Scale bar = 500 μm. (H) Enlargement of Fig G, showing the putative megaspores. Scale bar = 100 μm. (I) Enlargement of Fig H, showing the surface of a putative megaspore. Scale bar = 10 μm.

The collection includes mostly unbranched leafy axes (Figs 2A–2E and 3A–3C). Rare isotomously branched specimens have been found (Fig 2F). We believe that all the specimens belong to the same plant for the following reasons: (i) they co-occur at the locality, (ii) they all have similar size and aspect, (iii) one three-dimensionally preserved axis shows leaves identical to those of the impressions fossils (Fig 3), and (iv) there is no other plant in the fossiliferous beds. Primary and secondary tissues have been observed (Figs 4–6). Most specimens bear a distal strobilus (Figs 2A, 2B, 7 and 8). Micro- and putative megaspores were found in situ (Figs 9 and 10).

### Morphology of vegetative axis

The width of the vegetative axis (or of the vegetative portions of fertile axes) ranges from 1.5 mm to 5 mm. The leaves of the widest axes are borne in a low helix (Fig 3A–3C). Those of the vegetative portions of the fertile axes are borne in whorls or pseudowhorls (hereafter called pseudowhorls) (Figs 2A–2C and 7A, 7B). The pseudowhorls are 3–5 mm apart. The number of leaves per gyre or pseudowhorl is difficult to assess, but the specimen illustrated in Fig 3 suggests that the leaves are densely placed. No leaf bases have been observed.

#### Vegetative leaves

Vegetative leaves are inserted at a wide angle (45–90°) on the axis (Figs 2A–2C, 2F and 3). They are slightly decurrent (Fig 2B). They are at least 30 mm long and up to 1.7 mm wide (Fig 2A, 2C and 2D). Their width decreases slightly along their length. In most specimens, a deeply sunken groove is visible and presumably indicates the position of the midvein (Figs 2A, 2C, 2D, 3C and 7A). Leaf margin bears trichome-like appendages, up to 1 mm long and 0.1 mm wide in their proximal part (Fig 2D); trichome-like appendages are 5–10 mm apart along the leaf margin. Because of poor preservation, they are often hardly visible.

### Anatomy of axis

#### Primary xylem

Several three-dimensionally preserved axes have been observed under the SEM (Figs 4A–4D, 5B–5G and 6) or with reflected light under a stereoscopic microscope (Figs 4E, 4F and 5A). Some specimens include secondary xylem (Figs 4E, 4F and 5A). The primary xylem strand has a diameter of 1.0 to 1.8 mm in transverse section (Fig 4A, 4E and 4F), and shows 8–12 exarch protoxylem strands that appear as ridges around the metaxylem core. Halfway between two neighbouring protoxylem strands, a small group of tracheids, oval in cross-section, appears detached from the main vascular cylinder (Fig 4A–4C). Those groups of cells might represent the vascular supply (leaf trace) of the microphylls. Metaxylem cells are rounded in transverse section (Fig 4A–4D), 20–60 μm in diameter (Fig 4C and 4D). In longitudinal section (Fig 5B–5G), scalariform bars are visible; they are 4–7 μm thick. The pit aperture between the bars is 3–4 μm wide (Fig 5E–5G). At several places, the thickening bars are branched (Fig 5F). The pit apertures are crossed by several longitudinal narrow fimbrils also called Williamson’s striation (Fig 5F and 5G), 1–2 μm wide. In transverse section, the remains of these narrow fimbrils are also visible on the secondary wall of the tracheids (Fig 4D). Protoxylem cells are circular in cross section, ca. 7–20 μm in diameter (Fig 4A–4C). Protoxylem tracheids have annular/helical thickenings (Fig 5C and 5D).

#### Secondary xylem

The secondary xylem includes longitudinal tracheids (Fig 6A–6C) and rays (Figs 4E, 4F and 6B, 6D, 6E). The secondary xylem tracheids are 30–50 μm in diameter. Their secondary wall is scalariform, with thickening bars 3–5 μm thick and 3–5 μm apart. Often, the thickening bars are hollow (Fig 6C). Rays may be more than 100 cells high (Fig 6A and 6B). They include approximately rectangular thin-walled, presumably parenchymatous, cells, 20–50 μm high and 50–100 μm long (Fig 6A, 6B and 6E). Tracheid/ray (cross-field) pitting consists of ca. 20 rounded to oval pits, 5–10 μm high and wide (Fig 6D and 6E).

### Morphology of fertile axis

More than 50 fertile specimens have been collected. The best preserved are illustrated here (Figs 2A, 2B, 7A, 7B, 8A and 9A–9F). Fertile specimens often consist of a strobilus borne by an axis bearing whorled leaves. All are broken proximally.

#### Strobilus

Complete strobili (Figs 2A and 7A, 7B) are up to 56 mm long, and up to 7 mm wide, the upright part of the sporophylls excluded. They are composed of central axis bearing densely placed sporophylls. Sporophylls are disposed in a helix with 4 sporophylls per gyre or in pseudowhorls. The divergence angle between successive pseudowhorls is 90° (Fig 7C). The central part of all strobili is surrounded by the long upright portions of the fertile leaves. Specimens with microsporophylls have been found, as well as specimens with putative megasporophylls. However, it is not possible to assess if the plant has mono- or bisporangiate strobili.

#### Fertile leaf

Sporophylls are up to 45 mm long and 1.0–1.6 mm wide. They are inserted on the strobilus axis with an angle ranging from 45–90° (Figs 2A, 7A, 7B and 9D–9F). Sporophylls consist of a (sub)horizontal proximal portion (hereafter called pedicel) and a distal (sub)vertical lamina (Figs 7C and 9D–9F). The pedicel is devoid of lamina and of keel; it is approximately 2.0 to 3.5 mm long and 0.2–0.4 mm wide; it is presumably roughly triangular in cross-section (Fig 9E and 9F); it bears one sporangium on its adaxial surface. The distal lamina is recurved upward and up to 50 mm long. Trichome-like appendages, up to 0.5 mm long, are borne on their margin (Fig 2B and 2E); they are most generally badly preserved and hardly distinguishable. No ligule has been observed.

#### Sporangium

Rounded bodies are visible at many places on the adaxial surface of the sporophyl (Fig 8); they are interpreted as sporangia. Putative sporangia are globose, 1–2 mm high and wide (Fig 8A–8C). The dehiscence line has not been observed. They are possibly attached on a short stalk inserted distally on the pedicel of the sporophyll, near the angle between the latter and the upright lamina (Fig 8C).

#### Microspore

Numerous microspores are closely packed in the sporangia (Figs 9A–9C and 10A–10F). They are strongly affected by the diagenesis. The organic matter has been destroyed. Internal mineralized moulds have preserved some morphological details. Some specimens are preserved in tetrads (Fig 10B, 10D and 10E). The suture between the spores shows an elevated folded structure (Fig 10B and 10D). The microspore diameter is around 50 μm and varies little from one specimen to another. Their amb is subcircular. The trilete mark extends to the amb radius. Curvaturae are possibly present. A slightly prominent triangular area with concave sides is present at the proximal pole (Fig 10D). In the interradial and proximo-equatorial area, one specimen shows small parallel rugulae of 4–5 μm thick and apart, and 20–30 μm length in the interradial and proximo-equatorial area (Fig 10F). The distal face is smooth.

#### Putative megaspores

Rare specimens of megaspore-like rounded bodies have been observed (Figs 9D and 10G–10I). As are the microspores, the specimens are strongly affected by the diagenesis. The putative megaspores are 300–350 μm in diameter. Their shape is ovoid. The proximal face is not visible. The distal face shows parallel latitudinal convolute striae of more or less 1–2 μm width and 5 μm apart (Fig 10I).

## Comparative study

*Lilingostrobus* exhibits a unique set of characters among the Late Devonian–Early Carboniferous lycopsids; i. e. pseudoherbaceous habit (herbaceous in size but including limited secondary growth), pseudowhorls of long microphylls with trichomes on their margin, sporophylls including a (sub)horizontal pedicel without keel and alation and a long, upturned lamina, putative heterospory, solid protostele, secondary growth. No other previously published genus displays the same set of characters, which warrants our decision to include the plant described here in a new taxon. However, several genera discovered from Chinese localities share some morphological or anatomical features with *Lilingostrobus* and deserve more detailed comparisons (Table 1).

**Table 1:** Comparison among related Middle-Upper Devonian lycopsids from China.

	*Changxingia longifolia* *Changxingia* sp.	*Lilingostrobus* *chaloneri*	*Lobodendron* *fanwanense*	*Longostachys* *latisporophyllus*	*Sublepidodendron* *grabaui*	*Sublepidodendron* *songziense*	*Wuxia* *bistrobilata*
Locality	Changxing	Liling	Changxing	Longshan	Wuxi	Songzi	Wuxi
Province	Zhejiang	Hunan	Zhejiang	Hunan	Jiangsu	Hubei	Jiangsu
Formation	Wutong	Xikuangshan	Wutong	Yuntaikan	Wutung	Hsiehchingssu	Wutung
Age	Famennian	Famennian	Famennian	Givetian	Famennian	Famennian	Famennian
Axis							
Width	up to 20 mm	up to 5.0 mm	3.6–6.4 mm	10–35 mm	1.5 to 100 mm	Up to 70 mm?	up to 14 mm
Secondary growth	?	Yes	Yes	Yes	Yes	Yes	No
Leaf							
Shape	Linear	Linear	?	Linear	Linear/Acuminate	Linear or lanceolate	Linear
Length	18–25 mm	Up to 30 mm	?	20–70 mm	12 to > 60 mm	10–15 mm	Up to 63 mm
Width	0.5–1.2 mm	Up to 2.1 mm	?	6–10 mm	0.4 to 1.0 mm	0.7–1.2 mm	Up to 3 mm
Spines	No	Yes	?	Yes	No	No	Yes
N° leaves per gyre	?	?	?	Variable	6 to 14	?	6
Bisporangiate strobilus	No	?	?	?	No	No	No
Strobilus							
Length	NA	30 to 50 mm	?	30–225 mm	NA	NA	NA
Width	NA	Up to 14 mm	?	7–10 mm	NA	NA	NA
Monosporangiate strobili	Yes	?	?	?	Yes	Yes	Yes
Megasp. strobilus							
Length	Up to 50 mm	?	?	?	?	100–150 mm	Compact
Width	Up to 9.6 mm	?	?	?	?	6–9 mm	
Microsp. strobilus							
Length	?	?	?	?	Up to 160 mm	80–120 mm	105 mm
Width	?	?	?	?	Up to 10 mm	8–12 mm	20 mm?
Strobili distal only	Yes	?	?	Yes	Yes	Yes	No
Alation	Yes	No	?	Yes	Yes	Yes	No
Sporophyll							
Length	Up to 22 mm	Up to 45 mm	?	15–30 mm	Up to 14 mm	?	Up to 96 mm
Width	2.4–3.3 mm	1.6 mm	?	Up to 4.6 mm	Up to 2.5 mm?	?	2 to 3 mm
Pedicel position on axis	70°-90°	(Sub)horizontal	?	?	?	Horizontal	acutely inserted
Spines	No	Yes	?	Yes	No	No	Yes
Megaspore number	4?	?	?	4	?	?	4?
Megaspore diameter	Up to 910 μm	Up to 300 μm	?	Up to 2640 μm	Up to 1200 μm	Up to 550 μm	Up to 2 mm
References	[18,19]	This paper	[39]	[17]	[27,29]	[25,26,28]	[42]

*Changxingia longifolia* from the Late Devonian (Famennian) of Zhejiang Province [18,19], is a small-sized lycopsid assigned to the Dichostrobiles of the Isoetales *sensu* DiMichele and Bateman [12] on the basis of the possible presence of monosporangiate strobili. Its megasporophyll includes a pedicel (consisting of a keel and of horizontal alations), a heel and a short, gently abaxially curved lamina [18]. *Lilingostrobus* cannot be confused with this plant (Table 1).

*Lobodendron fanwanense* from the Late Devonian (Famennian) of Changxing (Zhejiang Province) [39], is based on anatomically preserved specimens only, so its external and reproductive morphologies are unknown. The plant consists of slender, dichotomously branched axes. Its stem includes a terete primary xylem strand surrounded by lobed secondary xylem, resulting from the activity of a possibly discontinuous cambium. Comparisons with this plant are difficult because the morphology and distribution of its leaves are not known. The secondary xylem of *Lododendron* is dissected into six to eight wedge-shaped radial arms [39] and hence looks different from that of *Lilingostrobus* which is in the form of a continuous layer around the primary xylem.

*Longostachys latisporophyllus* was discovered from the Middle Devonian (Givetian) of Hunan Province [17,40]. The species is described as being a small arborescent heterosporous plant, with helically disposed, simple, linear leaves bearing spiny appendages on their margin. The distal strobili are up to 22.5 cm long and 1 cm wide. The megasporophyll is spoon-like. The anatomy of the proximal parts of the plant includes a protostele surrounded by secondary xylem dissected into several wedge-shaped radial arms. In more distal parts of the plant including the strobilus axis, the primary xylem strand is a medullated siphonostele, with or without secondary xylem; when present, the secondary xylem forms a continuous thin layer. *Longostachys* and *Lilingostrobus* cannot be confused (Table 1).

The arborescent genus *Sublepidodendron* is common in Late Devonian and Early Carboniferous localities from Euramerica and China [26,29]. The genus shares some characteristics with *Lilingostrobus*, but, among the Chinese representatives of the genus, the species, *Sublepidodendron songziense* [26] and *Sublepidodendron grabaui* [27,29] show secondary growth. *S*. *grabaui* has been discovered from the Late Devonian Wutong Formation of the Jiangsu Province [27]. The trunk, branches and strobili of the plant are known. According to Wand and Xu [27], the secondary xylem of *S*. *grabaui* is found in the trunk only, where the primary xylem strand is a siphonostele. It is not the case for *Lilingostrobus*. Moreover, the habits of the two plants are different: the arborescent *Sublepidodendron grabaui* and the pseudoherbaceous *Lilingostrobus* cannot be confused. *Sublepidodendron songziense* occurs in the Late Devonian Xiejingsi and Hsiehchingssu Formation of the Hubei Province and the Wutong Formation of the Anhui Province, China [25,26,28,41]. The plant is characterized, among other features, by spirally inserted, small, vertically elongated leaf bases. Anatomical features of *S*. *songziense* include a siphonostele in all axes, with secondary xylem in the larger stems, where a thick periderm is present. These characters are not seen in *Lilingostrobus* (Table 1).

*Wuxia bistrobilata* from the Late Devonian (Famennian) of Wuxi (Jiangsu Province) [42], possesses isotomous branched axes and long leaves with a spiny margin. Leaves are borne in (pseudo?) whorls. Megasporangiate conelike structures are found at dichotomies of axes; they include large, densely placed leaves with enlarged bases, each bearing an adaxial megasporangium. Putative microsporangiate strobili have very narrow sporophylls, up to 55 mm long. They are positioned terminally on axes exhibiting the same leaf distribution as those bearing the megasporangiate conelike structures, but the conspecificity of the two types, admittedly probable, could not be unambiguously demonstrated. The putative microsporangiate strobili of *Wuxia* exhibit striking similarities with some specimens of *Lilingostrobus*, e.g., compare Berry et al. [42] with Figs 1 and 3. However, differences exist (Table 1). *Lilingostrobus* is overall much smaller than *Wuxia*: the axes, strobili and leaves of the former are roughly half the size of those of the latter. The proximal part of the megasporophyll of *Wuxia* is enlarged, while it is a narrow pedicel in *Lilingostrobus*. The megaspores of *Wuxia* are 2 mm in diameter; the putative megaspores of *Lilingostrobus* do not exceed 300 μm in diameter. No secondary growth has been described for *Wuxia*. All these differences, both in qualitative and quantitative characters, support the erection of a new genus for the specimens described in this paper. There is another important element that supports our decision to erect the new genus *Lilingostrobus*. According to the International Code of Nomenclature for algae, fungi, and plants (Melbourne Code) [43], (i) the type of a name of a genus is the type of a name of a species (Art. 10) [43] and (ii) the holotype of a name of a species is a single specimen (Art. 8) [43]. It means that the characters of a given genus are the same as the characters of the holotype of the type species of that genus. In this case, the holotype of the species *Wuxia bistrobilata* (which is the only species of the genus *Wuxia* and hence the type species) is a specimen with an intercalary cone-like structure, illustrated at fig 3b from Berry et al. [42], not a specimen with a terminal strobilus. Intercalary cone-like structures have never been found in *Lilingostrobus*. The possibility that incomplete strobili of *Lilingostrobus* are in fact not distally borne but represent intercalary cone-like structures has to be considered. However, we did not find any obviously intercalary cone-like structures in the whole material, which includes more than 50 fertile specimens. We therefore believe that our specimens consist only of axes with distal strobili, characterizing a genus clearly distinct from that represented by the type material of *Wuxia*. Additonally, the possibility that the assemblage of plants collectively called *Wuxia bistrobilata* actually includes two different plants cannot be dismissed.

**Fig 11:**
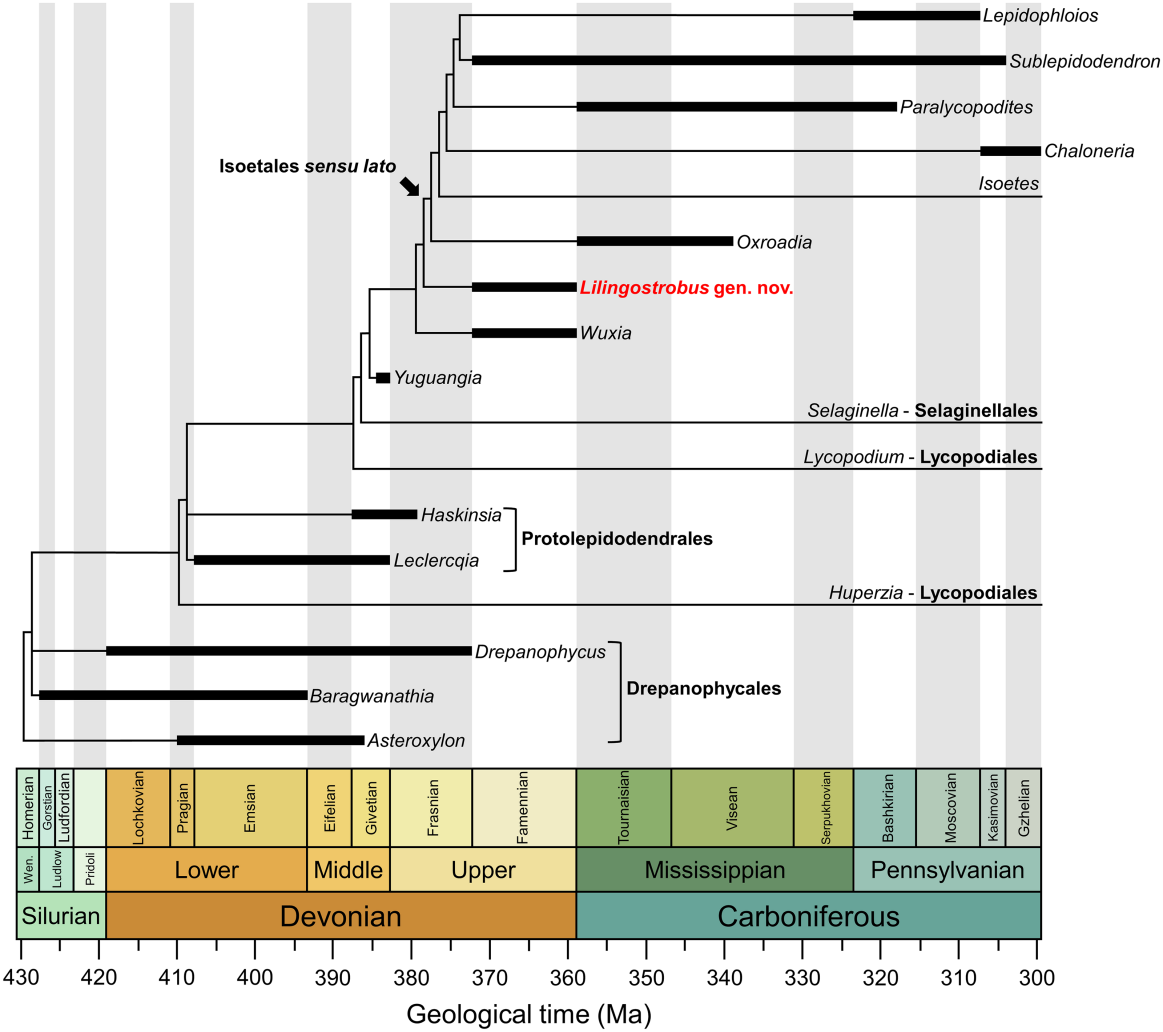
Time-scaled phylogeny of the earliest evolutionary relationships of lycopsids. Tree topology obtained from PAUP* 4.0. Time-scaled phylogeny plotted against stratigraphy via strap. Major clades are in bold. Black box indicates the temporal distribution of involved taxa. Raw data available from Supporting Information.

Phylogenetic results (Fig 11) shows that *Lilingostrobus* is in a sister-group relationship with the Isoetales *sensu* DiMichele and Bateman [12]. The only unambiguous synapomorphy of the clade “Isoetales *sensu* DiMichele and Bateman [12] + *Lilingostrobus*” is the presence of secondary xylem in the stem (character 13). Several other characters that are missing in *Lilingostrobus* (Table 1) are possible synapomorphies, namely a more or less circular root xylem shape (character 5), the presence of secondary xylem in root (character 6), the presence of rootlets (character 7), rhizomorphic rootlet anatomy (character 8), pseudobipolar growth (character 9), the presence of a 3-zoned cortex (character 16), the presence of a ligule located in a pit (character 23) and longitudinal dehiscence (character 33). More data on the putative rhizomorph and rootlets of *Lilingostrobus*, as well as on its fertile leaf, are definitely needed in order to decide if the genus is to be included within the Isoetales *sensu* DiMichele and Bateman [12].

## Discussion

### Habit

Even though their actual size is unknown because all are broken proximally, the specimens attributed to *Lilingostrobus* are of small size, being not longer than 10 cm and not wider than 5 mm. One slab shows 6 stems parallel to each other, with an interval of less than 10 mm between each (Fig 2B). Furthermore, the fossiliferous beds that have yielded *Lilingostrobus* include a large number of axes that never reach more than 10 mm in width. All these observations suggest that *Lilingostrobus* does not represent the distal parts of a larger plant, with possibly pendulous strobili, but that it was pseudoherbaceous, with a possible tufted habit. The large size of its strobili as compared to the small diameter of their subtending axis suggests the need for mutual lateral support for each individual stem and speaks in favour of this latter interpretation.

### Leaves

*Lilingostrobus* bears very long vegetative leaves and sporophylls, both being roughly of the same size (Figs 2A, 2B, 2D, 3B, 3C and 7A, 7B). All leaves possess a strong single midvein (Figs 3C and 7A, 7B) and trichomes along their lateral margin (Fig 2D and 2E). The presence of a strong midvein suggests that the leaves might have been rather rigid and that they might have provided additional support to the plant. The earliest land plants were leafless, and their sporangia have been shown to include a large number of stomata in their wall, as in the Lower Devonian genus *Hsüa* [44,45]. This has been interpreted as indicating that, in those early land plants devoid of leaves, the sporangium, beyond its spore-production function, had also a photosynthetic activity. It is assumed that, during the early evolution of leaves, the sporangia progressively lost their photosynthetic abilities and incorporated fewer stomata. The large size vein of the sporophylls of *Lilingostrobus* suggests that it included a large vascular bundle, which in turn suggests that the sporophylls had a high photosynthetic activity. It suggests that the sporophyll, beyond its commonly recognized protective function, played also an important role for the sporangium nutrition.

### Secondary xylem

*Lilingostrobus* had a most probably pseudoherbaceous habit and narrow stems. The presence of secondary xylem in such a small plant is puzzling. It can be hypothesized that the two main functions of the secondary xylem (water transport and support) were equally important for the plant. On the one hand, its narrow stems presumably needed additional support in relation with the presence of a large and compact distal strobilus with long leaves and, on the other hand, the presence of additional conducting cells of the secondary xylem presumably improved water transport towards the well-developed lamina of the sporophylls.

### Evolutionary considerations

In basal lycopsids such as the Middle Devonian Protolepidodendrales, e.g., *Minarodendron* [46,47], the vegetative and fertile leaves had more or less the same morphology; fertile leaves were dispersed amongst the vegetative leaves. These plants were all homosporous. From Givetian times onwards, a group of lycopsids evolved heterospory and strobili, structures where sporophylls are densely aggregated along a stem [16,17]. Simultaneously, sporophylls became morphologically distinct from the vegetative leaves. The sporophyll of strobilate lycopsids consists of a sporangium-bearing proximal pedicel and a distal, usually upturned, lamina. This position frequently leads to an overlapping of the sporophylls placed above in the same orthostichy. The lamina often extends downwards to form a heel or extension. The sporangium is borne on the adaxial surface of the pedicel. The pedicel may be laterally enlarged, as in *Wuxia*, and in this case the pedicel is accordingly described as spoon-shaped. In more advanced taxa, the pedicel of the megasporophyll is alate, which means that it acquires lateral foliar expansions interpreted as a protection layer for the megasporangium. The pedicel can also be downwardly extended into a keel. Only a few examples of Middle Devonian of strobilate lycopsids are known: *Mixostrobilus* [15], *Longostachys* [17,36], *Yuguangia* [16]. The latter is described as having bisporangiate strobili [16], which means that microsporophylls and megasporophylls are present in the same strobilus. Strobilate lycopsids diversified in the Late Devonian, and a large number of taxa are known. Representatives from China include the following genera: *Changxingia* [18,19], *Lepidostrobus* [20]; *Leptophloeum* [48], *Minostrobus* [21–24], *Sublepidodendron* [25–29], *Wuxia* [42]. The Late Devonian strobilate lycopsids had either bisporangiate strobili as in *Bisporangiostrobus*, from the Late Devonian of Pennsylvania [49] and *Clevelandodendron*, from the Late Devonian of Ohio [50] or monosporangiate strobili as in *Lepidostrobus* [20], *Minostrobus* [23] and *Sublepidodendron* [26]. The Late Devonian taxa with monosporangiate strobili are considered ancestral to the widespread Carboniferous Dichostrobiles *sensu* DiMichele and Bateman [12] [22].

*Lilingostrobus* possesses compact strobili; its sporophyll consists of a pedicel and a long, upturned lamina. We could not determine if *Lilingostrobus* had bi- or monosporangiate strobili. As a result, its systematic position remains uncertain. However, it is worth to note that *Lilingostrobus* exhibits a mixture of basal and of derived features. The basal features include: vegetative microphylls and sporophylls with roughly the same shape and length; absence of a heel formed by the proximal part of the lamina; pedicel non-alate, triangular in cross-section; sporangium (probably) attached to pedicel by a short stalk. Derived features include: heterospory, secondary growth, pedicel borne on the strobilus axis with a 45–90° angle (which compares closely with that of the Carboniferous Dichostrobiles).

Based on previous studies [8,12,16] and on our phylogenetic analysis, the evolutionary scenario for heterosporous lycopsids may have been the following. Heterospory first evolves in bisporangiate genera devoid of secondary growth such as *Yuguangia*. Secondary growth first occurred in the pseudoherbaceous *Lilingotrobus* and maybe, according to our phylogenetic analysis, in *Wuxia*. The arborescent habit then evolved, for example in *Longostachys*. The monosporangiate strobili character was finally acquired (in *Sublepidodendron* and other Dichostrobiles). The evolution of the megaspore number in the megasporangium is less easy to reconstruct. For instance, *Chaloneria* and *Paralycopodites* are phylogenetically close to the Dichostrobiles, even though the former produce many megaspores in each megasporangium and the latter only one. Similarly, the presence of four megaspores in the bisporangiate genera *Wuxia* and *Longostachys* is inconsistent with their position in our tree.

We could not evaluate the phylogenetic position of the recently described genus *Changxingia* [18,19] because the inclusion of the genus in the analysis resulted in poorly resolved topologies. Nevertheless, on the basis of the presence of monosporangiate strobili, *Changxingia* was assigned to the Dichostrobiles [18,19], which implies that the genus possessed secondary growth. This could not be demonstrated because no specimens, apart from spores, showed cellular preservation. Actually, the diminutive size of the stems of *Changxingia* suggests the absence of secondary growth and contrasts with the large size of the arborescent Dichostrobiles *Sublepidodendron* and *Lepidophloios*. This might be explained in different ways: (i) *Changxingia* represents the distal part of a larger, arborescent plant; (ii) as for *Lilingostrobus*, *Changxingia* possesses narrow stems with small quantities of secondary tissues; (iii) the “secondary growth character” is reversed in *Changxingia*.

## Conclusions

This paper is dedicated to the description of a new lycopsid, *Lilingostrobus chaloneri* gen. et sp. nov., collected from a Late Devonian (Famennian) locality from Hunan Province (South China). The plant adds to the already impressive diversity of the Devonian lycopsids in China. *Lilingostrobus* shows a so far unknown combination of characters: pseudoherbaceous, possibly tufted habit; vegetative microphylls with trichome-like appendages on their margin, borne in a low helix or in pseudowhorls; solid protostele; secondary xylem in a small amount; sporophylls with trichome-like appendages on their margin, aggregated in distal strobili. Microspores and putative megaspores have been found, but whether the plant has mono- or bisporangiate strobili is unknown.

The unusual suite of characters of *Lilingostrobus* helps to suggest the following evolutionary scenario for the Devonian heterosporous lycopsids. Heterospory first evolves in bisporangiate genera devoid of secondary growth such as *Yuguangia*. Secondary growth first occurred in the pseudoherbaceous *Lilingotrobus*. The arborescent habit then evolved, for example in *Longostachys*. The monosporangiate strobili character was finally acquired in *Sublepidodendron* and other Dichostrobiles. This scenario cannot be unambiguously demonstrated, because a range of characters is currently unknown in several taxa.

Despite the presence of basal characters in *Lilingostrobus*, the joint presence of heterospory and secondary growth in the plant strongly suggests that it is a stem (or an early) isoetalean. Additional specimens including proximal parts or better preserved strobili would be however needed to definitely assess the affinities of the plant.

## Supporting information

S1 Table Data matrix for the phylogenetic analysis. Raw data based on Xue [8]. Two taxa in bold (*Lilingostrobus* and *Wuxia*) have been added. Some modifications of raw coding in comparison with Xue [8] are shown in boxed numbers.
(PDF)

S2 Table List and temporal distribution of lycopsid genera involved in phylogenetic analysis. Absolute ages used for the time-scaled phylogeny appear in brackets. They are from the International Chronostratigraphic Chart (v2016/04). See S4 Text for supplementary references.
(PDF)

S3 Table Characters used in the phylogenetic analysis. Most are identical to those of Xue [8]. In bold: characters modified (2 to 4) in comparison with Xue [8].
(PDF)

S1 Text Data matrix (Nexus format) of PAUP analysis. (PDF)

S2 Text Tree file (Newick format) of *strap* analysis. (PDF)

S3 Text Age file (R package paleotree format) of *strap* analysis. (PDF)

S4 Text Supplementary references. (PDF)
