# Supplementary material for: Lilingostrobus chaloneri gen. et sp. nov., a Late Devonian woody lycopsid from Hunan, China
Source: PLoS One. 2018 Jul 11;13(7):e0198287. doi: 10.1371/journal.pone.0198287 (PMC6050970; doi:10.1371/journal.pone.0198287)
Supplement: S1 Table — Raw data based on Xue [8]. Two taxa in bold (Lilingostrobus and Wuxia) have been added. Some modifications of raw coding in comparison with Xue [8] are shown in boxed numbers. (PDF) [file pone.0198287.s001.pdf]

S1 Table. Data matrix for the phylogenetic analysis.

Raw data based on Xue [8]. Two taxa in bold (*Lilingostrobus* and *Wuxia*) have been added. Some modifications of raw coding in comparison with Xue [8] are shown in boxed numbers.

Taxa	Characters																															
	1	2	3	4	5	6	7	8	9	10	11	12	13	14	15	16	17	18	19	20	21	22	23	24	25	26	27	28	29	30	31	32	33
Asteroxylon	0	1	0	?	0	0	0	0	0	0	0	0	0	0	0	0	0	0	0	0	0	0	?	0	0	0	0	?	?	?	0	0	0
Baragwanathia	0	1	0	?	?	0	0	0	0	0	0	0	0	0	0	0	0	0	1	0	0	0	?	?	?	0	0	?	?	?	0	0	0
Chaloneria	1	0	2	0	0	1	1	1	1	1	1	1	1	1	1	1	1	1	1	0	1	1	1	1	1	1	1	0	1	1	2	0	1
Drepanophycus	0	1	0	?	?	0	0	0	0	0	0	0	0	0	0	0	0	0	1	0	0	0	?	0	?	0	0	?	?	?	0	0	0
Haskinsia	0	1	0	?	?	0	0	0	0	1	0	0	0	1	?	1	0	0	1	0	0	0	?	1	0	0	0	?	0	0	1	0	?
Huperzia	0	1	0	?	1	0	0	0	0	0	0	0	0	1	?	0	0	0	1	0	0	0	?	1	0	0	0	?	0	0	0	0	0
Isoetes	1	0	2	0	0	1	1	1	1	1	0	0	1	1	?	1	0	?	1	0	?	1	1	1	?	1	?	0	1	1	2	1	?
Leclercqia	0	1	0	?	?	0	0	0	0	1	0	0	0	1	?	0	0	0	1	1	0	1	0	1	0	0	0	?	0	0	2	0	1
Lepidophloios	1	2	2	1	0	1	1	1	1	1	1	1	1	1	1	1	1	1	1	0	1	1	1	1	1	1	1	1	1	1	2	0	1
Lilingostrobus	**1**	**1**	**?**	**?**	**?**	**?**	**?**	**?**	**?**	**1**	**0**	**0**	**1**	**1**	**1**	**?**	**?**	**?**	**?**	**0**	**0**	**?**	**?**	**1**	**1**	**1**	**1**	**?**	**1**	**?**	**1**	**?**	**?**
Lycopodium	1	1	0	?	1	0	0	0	0	0	0	0	0	1	?	0	0	0	1	0	0	0	?	1	1	0	1	?	0	0	1	0	0
Oxroadia	1	1	1	1	0	1	1	1	1	1	0	0	1	1	1	1	0	0	1	0	0	1	1	1	1	?	1	0	1	1	2	0	1
Paralycopodites	1	2	1	1	0	1	1	1	1	1	1	1	1	1	1	1	1	1	1	0	1	1	0	1	1	1	1	0	1	1	2	0	1
Selaginella	1	1	0	?	1	0	0	0	0	1	0	0	0	1	?	0	0	0	1	0	0	1	0	1	1	1	1	0	1	0	1	0	0
Sublepidodendron	1	2	2	1	?	?	1	1	1	1	1	1	1	1	1	?	1	1	1	0	0	?	?	1	1	1	1	1	1	1	2	0	1
Wuxia	**1**	**1**	**?**	**?**	**?**	**?**	**?**	**?**	**?**	**1**	**0**	**0**	**0**	**1**	**1**	**?**	**0**	**?**	**1**	**0**	**0**	**?**	**?**	**1**	**1**	**1**	**0**	**1**	**1**	**?**	**?**	**?**	**?**
Yuguangia	1	1	?	?	?	?	?	?	?	1	0	0	0	1	0	0	0	1	1	0	0	1	0	1	1	1	1	0	1	1	1	0	0
