# Supplementary material for: Lilingostrobus chaloneri gen. et sp. nov., a Late Devonian woody lycopsid from Hunan, China
Source: PLoS One. 2018 Jul 11;13(7):e0198287. doi: 10.1371/journal.pone.0198287 (PMC6050970; doi:10.1371/journal.pone.0198287)
Supplement: S2 Table — Absolute ages used for the time-scaled phylogeny appear in brackets. They are from the International Chronostratigraphic Chart (v2016/04). See S4 Text for supplementary references. (PDF) [file pone.0198287.s002.pdf]

S2 Table. List and temporal distribution of lycopsid genera involved in phylogenetic analysis.

Absolute ages used for the time-scaled phylogeny appear in brackets. They are from the International Chronostratigraphic Chart (v2016/04). See S4 Text for supplementary references.

Taxa	Temporal distribution (Ma)	References
Asteroxylon	Pragian (but not earliest)–early Givetian (410.0-386.0)	[50–52]
Baragwanathia	Gorstian–Emsian (427.4-393.3)	[6,53,54]
Chaloneria	Kasimovian–Gzhelian (307.0-298.9)	[55]
Drepanophycus	Lochkovian–Frasnian (419.2-372.2)	[56,57]
Haskinsia	Givetian–early Frasnian (387.7-379.2)	[58]
Huperzia	Present (0.0)	Extant
Isoetes	Present (0.0)	Extant
Leclercqia	Emsian–Givetian (407.6-382.7)	[59,60]
Lepidopholios	Bashkirian–Moscovian (323.2-307.0)	[61]
Lilingostrobus	Famennian (372.2-358.9)	This study
Lycopodium	Present (0.0)	Extant
Oxroadia	Tournaisian–middle Visean (358.9-338.9)	[62,63]
Paralycopodites	Tournaisian–late Bashkirian (358.9-317.9)	[64,65]
Selaginella	Present (0.0)	Extant
Sublepidodendron	Famennian–Kasimovian (372.2-303.7)	[26]
Wuxia	Famennian (372.2-358.9)	[39]
Yuguangia	late Givetian (384.4-382.7)	[16]
