# Supplementary material for: Lilingostrobus chaloneri gen. et sp. nov., a Late Devonian woody lycopsid from Hunan, China
Source: PLoS One. 2018 Jul 11;13(7):e0198287. doi: 10.1371/journal.pone.0198287 (PMC6050970; doi:10.1371/journal.pone.0198287)
Supplement: S3 Table — Most are identical to those of Xue [8]. In bold: characters modified (2 to 4) in comparison with Xue [8]. (PDF) [file pone.0198287.s003.pdf]

S3 Table. Characters used in the phylogenetic analysis.
Most are identical to those of Xue [8]. In bold: characters modified (2 to 4) in comparison with Xue [8].

N°	Characters
1	Growth of individuals : 0=indeterminate ; 1=determinate.
2	**Aerial axes: 0=aerial axis unbranched; 1=isotomous; 2=more or less pseudomonopodial.**
3	**Rhizomorph: 0=absent; 1=radially symmetrical; 2: bilaterally symmetrical.**
4	**Rhizomorph: 0=unbranched; 1=branched.**
5	Root xylem outline in transverse section: 0=more or less circular; 1=C- or U-shaped.
6	Secondary xylem in root: 0=absent; 1=present.
7	Rootlet: 0=absent; 1=present.
8	Rootlet anatomy: 0=simple; 1=rhizomorphic.
9	Growth habit: 0=monopolar; 1=pseudobipolar.
10	Stem xylem outline in transverse section: 0=stellate; 1=more or less terete.
11	Xylem strand structure: 0=solid; 1=medullated.
12	Longitudinal ridges of protoxylem: 0=distinguishable; 1=indistinguishable.
13	Secondary xylem in stem: 0=absent; 1=present.
14	Metaxylem tracheid pitting: 0=absent; 1=present.
15	Interbar material between tracheid thickenings: 0=pitlet sheet; 1=Williamson’s striation.
16	Cortex tissue: 0=two-zoned; 1=three-zoned.
17	Leaf cushions: 0=absent; 1=present.
18	Shape of leaf bases or cushions: 0=more or less circular; 1=longitudinally elongated.
19	Leaf vein: 0=absent; 1=present.
20	Leaf shape: 0=simple; 1=forked.
21	Leaf abscission: 0=absent; 1=present.
22	Ligule: 0=absent; 1=present.
23	Ligule position: 0=superficial; 1=in pit.
24	Sporophyll: 0=absent; 1=present.
25	Sporophyll distribution: 0=loosely dispersed on stem; 1=aggregated in strobilus.
26	Heteropspory: 0=absent; 1=present.
27	Fertile structure: 0=intercalary; 1=terminal.
28	Strobilus: 0=bisporangiate; 1=monosporangiate.
29	Sporophyll shape: 0=more or less unmodified; 1= horizontal pedicel and upturned lamina.
30	Sporangium attachment: 0=stalked; 1=unstalked or attached by a pad or subarchesporial tissue.
31	Sporangium shape: 0=flattened; 1=more or less spherical; 2=radially elongated.
32	Sporangium dehiscence: 0=absent; 1=present.
33	Dehiscence: 0=transverse; 1=longitudinal.
