# Supplementary material for: Lilingostrobus chaloneri gen. et sp. nov., a Late Devonian woody lycopsid from Hunan, China
Source: PLoS One. 2018 Jul 11;13(7):e0198287. doi: 10.1371/journal.pone.0198287 (PMC6050970; doi:10.1371/journal.pone.0198287)
Supplement: S1 Text — (PDF) [file pone.0198287.s004.pdf]

```
#nexus

begin data;
dimensions ntax=17 nchar=33;
format
symbols="012";

matrix



Asteroxylon          010?000000000000000000?0000???000
Baragwanathia        010??00000000000001000???00???000
Chaloneria           102001111111111111101111111011201
Drepanophycus        010??00000000000001000?0?00???000
Haskinsia            010??000010001?1001000?1000?0010?
Huperzia             010?1000000001?0001000?1000?00000
Isoetes              1020011111001 1?10?10?111?1?01121?
Leclercqia           010??000010001?000110101000?00201
Lepidophloios        122101111111111111101111111111201
Lilingostrobus       11???????100111????00??1111?1?1??
Lycopodium           110?1000000001?0001000?1101?00100
Oxroadia             1111011111001111001001111?1011201
Paralycopodites      121101111111111111101101111011201
Selaginella          110?1000010001?000100101111010100
Sublepidodendron     1221??111111111?11100??1111111201
Wuxia                11???????100011?0?100??111011????
Yuguangia            11???????100010001100101111011100

;

endblock;
begin command;
;
endblock;
begin PAUP;
SET OUTROOT=monophyl;
endblock;
```
