# Supplementary material for: Lilingostrobus chaloneri gen. et sp. nov., a Late Devonian woody lycopsid from Hunan, China
Source: PLoS One. 2018 Jul 11;13(7):e0198287. doi: 10.1371/journal.pone.0198287 (PMC6050970; doi:10.1371/journal.pone.0198287)
Supplement: S2 Text — (PDF) [file pone.0198287.s005.pdf]

Tree topology data:

((((((((((((((Lepidophloios,Sublepidodendron),Paralycopodites),Chaloneria),Isoetes),Oxroadia
),Lilingostrobus),Wuxia),Yuguangia),Selaginella),Lycopodium),Haskinsia,Leclercqia),Huper
zia),Drepanophycus,Baragwanathia),Asteroxylon);
