# Supplementary material for: Lilingostrobus chaloneri gen. et sp. nov., a Late Devonian woody lycopsid from Hunan, China
Source: PLoS One. 2018 Jul 11;13(7):e0198287. doi: 10.1371/journal.pone.0198287 (PMC6050970; doi:10.1371/journal.pone.0198287)
Supplement: S3 Text — (PDF) [file pone.0198287.s006.pdf]

Age data:

	FAD	LAD
Asteroxylon	410.0	386.0
Baragwanathia	427.4	393.3
Chaloneria	307.0	298.9
Drepanophycus	419.2	372.2
Haskinsia	387.7	379.2
Huperzia	0.0	0.0
Isoetes	0.0	0.0
Leclercqia	407.6	382.7
Lepidophloios	323.2	307.0
Lilingostrobus	372.2	358.9
Lycopodium	0.0	0.0
Oxroadia	358.9	338.9
Paralycopodites	358.9	317.9
Selaginella	0.0	0.0
Sublepidodendron	372.2	303.7
Wuxia	372.2	358.9
Yuguangia	384.4	382.7
