# Supplementary material for: Lilingostrobus chaloneri gen. et sp. nov., a Late Devonian woody lycopsid from Hunan, China
Source: PLoS One. 2018 Jul 11;13(7):e0198287. doi: 10.1371/journal.pone.0198287 (PMC6050970; doi:10.1371/journal.pone.0198287)
Supplement: S4 Text — (PDF) [file pone.0198287.s007.pdf]

Supplementary references

50. Kräusel R, Weyland H. Beitrage zur Kenntnis der Devonflora II. Uber Material und Fundorte. Abh Senckenb Naturforsch Ges. 1926; 40: 114–155.

51. Lyon AG. The probable fertile region of *Asteroxylon mackei* K. and L. Nature. 1964; 203: 1082–1083. https://doi.org/10.1038/2031082b0

52. Wellman CH. Spore assemblages from the Lower Devonian 'Lower Old Red Sandstone' deposits of the Rhynie outlier, Scotland. Trans R Soc Edinb Earth Sci. 2006; 97: 167–211. https://doi.org/10.1017/S0263593300001449

53. Lang WH, Cookson IC. On a flora, including vascular land plants, associated with *Monographus*, in rock of Silurian age, from Victoria, Australia. Phil Trans R Soc Lond. 1935; 224: 421–449. https://doi.org/10.1098/rstb.1935.0004

54. Hueber FM. A new species of *Baragwanathia* from the Sextant Formation (Emsian) Northern Ontario, Canada. Bot J Linn Soc. 1983; 86: 57–79. https://doi.org/10.1111/j.1095-8339.1983.tb00717.x

55. Pigg KB, Rothwell GW. *Chaloneria* gen. nov.; heterosporus lycophytes from the Pennsylvanian of North America. Bot Gaz. 1983; 144: 132–147. https://doi.org/10.1086/337354

56. Grierson JD, Banks HP. Lycopods of the Devonian of New York State. Palaeontogr Am. 1963; 4: 220–295.

57. Schweitzer H-J. Uber *Drepanophycus spinaeformis* Göppert. Bonner Paläobotanische Mitteilungen. 1980; 7: 1–29.

58. Cingolani CA, Berry CM, Morel E, Tomezzoli R. Middle Devonian lycopsids from high southern palaeolatitudes of Gondwana (Argentina). Geol Mag. 2002; 139: 641–649. https://doi.org/10.1017/S0016756802006957

59. Banks HP, Bonamo PM, Grierson JD. *Leclercqia complexa* gen. et sp. nov., a new lycopod from the late Middle Devonian of eastern New York. Rev Palaeobot Palynol. 1972; 14: 19–40. https://doi.org/10.1016/0034-6667(72)90005-X

60. Wellman CH, Gensel P, Taylor WA. Spore wall ultrastructure in the early lycopsid *Leclercqia* (Protolepidodendrales) from the Lower Devonian of North America: evidence

for a fundamental division in the lycopsids. Am J Bot. 2009; 96: 1849–1860. https://doi.org/10.3732/ajb.0800422

61. DiMichele DM. Arborescent lycopods of Pennsylvanian age coals: *Lepidophloios*. Palaeontogr Abt B. 1979; 171: 57–77. http://www.schweizerbart.de/papers/palb/detail/171/72262/Arborescent_lycopods?l=EN

62. Rex GM, Scott AC. The Sedimentology, Palaeoecology and Preservation of the Lower Carboniferous Plant Deposits at Pettycur, Fife, Scotland. Geol Mag. 1987; 124: 43–66. https://doi.org/10.1017/S0016756800015776

63. Bateman RM, Scott AC. A reappraisal of the Dinantian floras at Oxroad Bay, East Lothian, Scotland. 2. Volcanicity, palaeoenvironments and palaeoecology. Trans R Soc Edinb. 1990; 81: 161–194. https://doi.org/10.1017/S0263593300005228

64. Scott AC, Galtier J, Clayton G. Distribution of anatomically-preserved floras in the Lower Carboniferous in Western Europe. Trans R Soc Edinb. 1984; 75: 311–340. https://doi.org/10.1017/S026359330001395X

65. Galtier J. Coal-ball floras of the Namurian-Westphalian of Europe. Rev Palaeobot Palynol. 1997; 95: 51–72. https://doi.org/10.1016/S0034-6667(96)00027-9
